# Scientific Applications of Distributed Acoustic Sensing: State-of-the-Art Review and Perspective

**DOI:** 10.3390/s22031033

**Published:** 2022-01-28

**Authors:** Boris G. Gorshkov, Kivilcim Yüksel, Andrei A. Fotiadi, Marc Wuilpart, Dmitry A. Korobko, Andrey A. Zhirnov, Konstantin V. Stepanov, Artem T. Turov, Yuri A. Konstantinov, Ivan A. Lobach

**Affiliations:** 1 Prokhorov General Physics Institute RAS, St. Vavilova, 38, GSP-1, 119991 Moscow, Russia; bggorshkov@gmail.com; 2 Petrofiber, LLC, Klinsky Proezd 7, 301664 Novomoskovsk, Russia; 3 Electrical and Electronics Engineering Department, Izmir Institute of Technology, Urla, Izmir TR-35430, Turkey; kivilcimyuksel@iyte.edu.tr; 4 S.P. Kapitsa Research Institute of Technology, Ulyanovsk State University, 42 Leo Tolstoy Street, 432970 Ulyanovsk, Russia; korobkotam@rambler.ru; 5 Ioffe Physical-Technical Institute of the RAS, 26 Polytekhnicheskaya Street, 194021 St. Petersburg, Russia; 6 Electromagnetism and Telecommunication Unit, Faculty of Engineering, University of Mons, Boulevard Dolez 31, 7000 Mons, Belgium; marc.wuilpart@umons.ac.be; 7 Bauman Moscow State Technical University, 2-nd Baumanskaya 5-1, 105005 Moscow, Russia; a.zh@bmstu.ru (A.A.Z.); stkv@bmstu.ru (K.V.S.); 8 Kotelnikov Institute of Radioengineering and Electronics of RAS, Mokhovaya 11-7, 125009 Moscow, Russia; 9 Perm Federal Research Center of the Ural Branch of the Russian Academy of Sciences (PFRC UB RAS), 13a Lenina St., 614990 Perm, Russia; artemtur442@gmail.com (A.T.T.); yuri.al.konstantinov@ro.ru (Y.A.K.); 10 General Physics Department, Applied Mathematics and Mechanics Faculty, Perm National Research Polytechnic University, Prospekt Komsomolsky 29, 614990 Perm, Russia; 11 Institute of Automation and Electrometry, Siberian Branch, Russian Academy of Sciences, 630090 Novosibirsk, Russia; lobach@iae.nsk.su

**Keywords:** optical reflectometry, fiber optic sensors, distributed acoustic sensing (DAS)

## Abstract

This work presents a detailed review of the development of distributed acoustic sensors (DAS) and their newest scientific applications. It covers most areas of human activities, such as the engineering, material, and humanitarian sciences, geophysics, culture, biology, and applied mechanics. It also provides the theoretical basis for most well-known DAS techniques and unveils the features that characterize each particular group of applications. After providing a summary of research achievements, the paper develops an initial perspective of the future work and determines the most promising DAS technologies that should be improved.

## 1. Introduction

The development of modern science is inconceivable without the constant improvement of research equipment and the design of novel tools providing new knowledge about the world around us. One of the most critical issues in this development process is the implementation of existing modern research equipment and methods in those areas of science where they have not yet been exploited. One of the most modern families of measuring instruments are fiber-based sensors, intelligibly presented by Fang et al. [1]. The conventional telecom optical fibers but also some specialist ones can indeed be used for sensing purposes. The advantages of fiber-optic sensors in contrast to electrical ones are that they are lightweight, small in size, have an electricity-free sensing element, and are immune to electromagnetic interference and aggressive media [2]. As a result, they can be used in an explosive atmosphere, combustible mixtures [3], and strong electromagnetic fields. Distributed fiber sensors have a special place among fiber optic sensors and represent a truly new technological milestone in metrology and scientific research. Their features and advantages have been demonstrated by A. Hartog in [4]. They have been rapidly adopted in new areas of research and as a result they have helped to solve many practical problems. The reason is quite simple: an optical fiber or cable can be placed around the entire perimeter or volume of the object under study and can obtain the necessary information about it in a distributed (spatially resolved) manner. Such fiber systems consist of a sensing element—a conventional optical fiber—and an interrogator for the generation of an optical probe signal and an analysis of the signal scattered in the fiber-based sensor [1,4]. Local measurement of physical parameters using these sensors provides a unique opportunity to monitor distributed structures. For this reason, distributed sensors are a promising alternative to the arrays of pointwise sensors. A single fiber optic cable can potentially replace thousands of them, greatly simplifying both the measurement setup itself and the interrogation process [4,5,6]. There are three main types of scattering in an optical fiber, which are utilized in distributed sensing—Rayleigh, Brillouin, and Raman [1,4,7]. The characteristics (such as amplitude, frequency, polarization) of the scattered radiation can depend on the physical action to be monitored.

For example, the work of distributed fiber sensors applying the Brillouin scattering is based on the fact that the Brillouin frequency shift (BFS) (peak frequency shift of the Brillouin gain spectrum (BGS) or Brillouin loss spectrum (BLS)) linearly depends on the distributed information (temperature or strain) [8]. These sensors can be realized in the time, frequency, and correlation domains [9,10,11,12]. Among them, Brillouin Optical Time-Domain Analysis is a typical time-domain Brillouin sensor, where stimulated Brillouin scattering (SBS) interaction takes place between a pulsed pump and a counter-propagating continuous probe wave when their frequency offset falls in the BGS or BLS [13]. 

Distributed fiber sensors are used to measure the distribution of temperature [14,15,16,17,18,19,20,21], stress/deformations [22,23,24], or vibro-acoustic properties [25,26,27,28,29,30] of various objects. The last application allows more information about the investigated object to be obtained in contrast to the static measurement of temperature or stress. In this case researchers obtain not only the magnitude of the impact, but also the frequency of its variation. The acoustic field emitted by an object/event (i.e., its sound image) provides an important piece of information about it, as almost everything that happens has, in fact, its own sound [31]. Fiber-based Distributed Acoustic Sensors (DAS) are powerful instruments for the analysis of acoustic fields with a sweep along the longitudinal coordinate i.e., a DAS system performs as a distributed microphone. DAS technology has been rapidly commercialized and is found in many applications in the industry. The most popular DAS application is perimeter security [32,33,34] including in airports [35], railways [36], power plants, and other vital areas. The typical parameters of the DAS security system are a maximum fiber length od up to 100 km with an accuracy of fault detection up to 10 m, a frequency sensitivity range from 0.5 Hz to 20 kHz (depending on fiber length) with deformation sensitivity < 1 nε. Another important industrial DAS application is geophysics [37,38]. This application was presented 10 years ago [39]. Since then, the number of geophysical studies with DAS has been constantly growing. The DAS technique is used for seismic data acquisition in vertical seismic profiling [40,41,42,43,44], microseismic measurements [45,46], and hydraulic fracturing monitoring and diagnostics [47,48,49]. DAS can be used for simultaneous land near-surface characterization and subsurface imaging in boreholes [50] so that they can substitute geophones that are well-known and widespread in industry. A large dynamic range at low frequencies is one of the advantages of DAS over geophones. The constant development of DAS systems has led to an increase in their popularity, providing unique solutions for some specific problems. For instance, these systems allow the observation of ocean and solid earth phenomena in marine geophysics, which has been described by Hartog et al. in [51]. In this case, existing subsea cables (dark fibers) can be used as sensing elements. DAS also provide the opportunity to track sea-state dynamics during a storm cycle. One more industrial application of DAS is the analysis of the material (in buildings, structures, vehicles, etc.) crack formation process. DAS can perform dynamic measurements of distributed strains, deflections, and crack widths in reinforced concrete structures as well [52,53]. The ability to properly examine existing reinforced concrete structures prevents expensive reconstruction or replacement and helps future designs to be optimized.

There are lots of essential reviews devoted to the technical applications of DAS systems [54,55,56]. In these reviews the general advances of DAS are described [56]: good time and space resolution, big spatial coverage, nice environment adaptability, convenient implementation, and others. Moreover, it is noted that the economic benefits of using distributed acoustic sensors need to be assessed on a case-by-case basis. This becomes more likely when, instead of an array of expensive sensors or microphones, a single distributed or quasi-distributed sensor on an optical fiber can be used. The impressive price of such systems also plays a significant role in the selection (these aspects will be discussed in detail in the conclusion). Other details on which the published reviews are focused on are the setups and technical details, and applications in industry, but most of them suffer from the lack of information about the scientific applications. By “scientific application”, the authors mean that the measurement device (commercially available or not) is used as a scientific instrument, i.e., as an apparatus that could lead to better knowledge of a physical or biological phenomenon.

A group of purely scientific or “exotic” DAS applications have also been studied in the scientific literature of various disciplines. This is due to the fact that sound plays an important role in nature and acoustic data provide a wealth of information about the environment and what is happening in it. Such data may result from geophysical studies, the sound image of processes in a mine or borehole, the sound of forests, marine fauna, the acoustic snapshot of highways, etc. The literature review covers all the application areas mentioned above with an outline of the most relevant outcomes as well as an assessment of the prospects of research directions. The fundamentals of distributed acoustic sensing and the advantages and limitations are also provided in the theoretical background section.

## 2. Theoretical Background (Andrei A. Fotiadi, Dmitry A. Korobko, Kivilcim Yüksel, and Marc Wuilpart)

### 2.1. A Brief History of Fiber Optic Acoustic Sensors

The study of acoustic vibration, its effect on the characteristics of the medium, and how the light propagates in it has been carried out by many scientists since the early 20th century [57].

The first acoustic sensors based on Rayleigh backscattering in optical fibers appeared in the literature after the sufficiently low-loss optical fiber was demonstrated [58]. These sensors then began to rapidly gain popularity in many fields, such as in perimeter protection [59], underwater acoustic sensing (using fiber Bragg gratings) [60], the structural integrity monitoring of composite materials (using a Fabry–Perot interferometer and a cavity at the end of an optical fiber) [61], partial discharge monitoring of transformers [62], and control of hydrocarbon spills (using a polymer-clad optical fiber) [63].

Nowadays, such sensors are also widely used in geophysics and traffic control. Applications in natural sciences and culture have begun to develop. The new applications have emerged due to the ability of such sensors to meet some unique needs and the more economical total cost of such systems.

### 2.2. Mathematical Formalism for Distributed ϕ-OTDR Sensing

The distributed phase-sensitive time–domain sensing employs the coherent light backscattered from the sensing fiber for the detection and analysis of perturbations induced by an external physical field on the sensing fiber. Probe pulses emitted from a highly coherent laser source (the coherence length is much longer than the pulse width) are injected into the sensing fiber one by one and backscattering signals induced by each of them are recorded at the fiber input and then compared to localize and evaluate the external forces applied to the sensing fiber during such monitoring. In this section, we present the mathematical formalism underlying the basic principles of ϕ-OTDR sensing.

**Figure 1:**
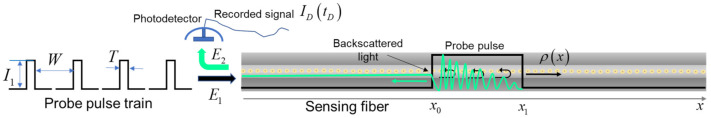
An illustration of a ϕ-OTDR sensor operation. Here $E_{1}$ and $E_{2}$ are the probe pulse and backscattering field amplitudes; $I_{1}$, T, and W are the pulse’s peak power, duration, and period; x is the coordinate along the sensing fiber, $\rho \left(x\right)$ is the relative reflectivity of the Rayleigh backscattering centers distributed along the fiber length.

Let the probe pulse be injected into the sensing fiber with low linear losses $\alpha \left(x\right)$ distributed over the fiber length (See Figure 1). At the moment t=0, i.e., at t=0 the leading pulse edge is located at the fibre input x=0. The pulse is assumed to be of rectangular shape, $I_{1}$ is the peak pulse intensity, and T is the pulse duration. The pulse propagates through the fiber and produces Rayleigh backscattering caused by multiple reflections of the pulse on nonuniformities of the refractive index frozen into the fiber during its manufacturing. These refractive index nonuniformities are modeled here as the Rayleigh reflection centers with the relative reflectivity $∼\rho \left(x\right)$ randomly distributed over the whole fiber length. When at a specific moment $t_{0}+T$, the pulse leading edge obtains the fiber point $x_{1}=\frac{c}{n}\left(t_{0}+T\right)$, the pulse trailing edge is at the point $x_{0}=\frac{c}{n}t_{0}$. The light backscattered on the distributed reflection centers forms an interference pattern inside the pulse providing the intensity at the point of pulse trailing edge referred here to as $I_{2}\left(t_{0},x_{0}\right)$. Once formed, this intensity is transmitted to the fiber input without any further changes. The complex amplitude $E_{2}\left(t_{0}+T,x_{0}\right)$ of the electric field associated with the intensity $I_{2}\left(t_{0}+T,x_{0}\right)$ is expressed as a superposition of all backscattered fields that are generated by the pulse in the preceding time and obtain the point $x_{0}$ exactly at the moment ($t_{0}+T$). 

The backscattering events satisfying this criterion occur in time between $t_{0}+\frac{T}{2}$ (when the leading pulse edge generates the backscattering signal at the fiber point $x_{0}+\frac{c}{n}\frac{T}{2}$) and $t_{0}+T$ (when the pulse trailing edge generates the backscattering signal at the fiber point $x_{0}$). Therefore, the field $E_{2}\left(t_{0}+T,x_{0}\right)$ reads as:

(1) $$E_{2}\left(t_{0}+T,x_{0}\right)=\int_{0}^{\frac{T}{2}}E_{1}\left(t_{0}+T-\tau ,x_{0}+\frac{c}{n}\tau \right)\rho \left(x_{0}+\frac{c}{n}\tau \right)d\tau$$

where $E_{1}\left(t,x\right)$ is the complex amplitude of the probe pulse.

The probe pulse is assumed to be a piece of a monochromatic optical wave at the frequency $\omega_{0}=2\pi f_{0}=\frac{2\pi }{\lambda_{0}}\frac{c}{n}$, where $\lambda_{0}$ is the laser wavelength. Moreover, this pulse propagates through the single-mode sensing fiber with the group velocity of $\frac{c}{n}$:

(2) $$\left[\begin{array}{l} E_{1}\left(t,x\right)=\sqrt{\gamma \left(x\right)I_{1}}\exp\left\{i\omega_{0}\left(t-\frac{n}{c}x\right)\right\}\;for\;t\in \left[\frac{n}{c}x;\;\frac{n}{c}x+T\right],\;x\in \left[0,L\right]\\ E_{1}\left(t,x\right)=0\;otherwise \end{array}\right.$$

where the integrated loss factor is expressed as

(3) $$\gamma \left(x\right)=\exp\left(-\int_{0}^{x}\alpha \left(x^{\prime }\right)dx^{\prime }\right)$$

Substituting Equation (2) into Equation (1) gives an expression for the backscattered signal amplitude:

(4) $$E_{2}\left(t_{0}+T,x_{0}\right)=\sqrt{\gamma \left(x_{0}\right)I_{1}}\exp\left\{i\left(\omega_{0}\left(t_{0}+T-\frac{n}{c}x_{0}\right)\right)\right\}\int_{0}^{\frac{T}{2}}\exp\left\{-i2\omega_{0}\tau \right\}\rho \left(x_{0}+\frac{c}{n}\tau \right)d\tau$$

and, thereafter, for its intensity:

(5) $$I_{2}\left(t_{0}+T,x_{0}\right)=\gamma \left(x_{0}\right)I_{1}\int_{0}^{\frac{T}{2}}\int_{0}^{\frac{T}{2}}\exp\left\{-i2\omega_{0}\left(\tau -\tau^{\prime }\right)\right\}\rho \left(x_{0}+\frac{c}{n}\tau \right)\rho^{*}\left(x_{0}+\frac{c}{n}\tau^{\prime }\right)d\tau \;d\tau^{\prime }$$

The backscattered light intensity formed at the pulse trailing edge point is transmitted then by the fiber to the fiber input point x=0 and is recorded by the photodetector at the moment $t_{D}=\left(2t_{0}+T\right)$. One can see from Equation (4) that the detected intensity $I_{D}\left(t_{D}\right)$ is contributed by the light backscattered from the points within the fiber segment $\left[x_{0},x_{0}+\frac{c}{n}\frac{T}{2}\right]\equiv \left[x_{D}-\frac{\Delta }{2},x_{D}+\frac{\Delta }{2}\right]$. The mean interval point

(6) $$x_{D}=x_{0}+\frac{c}{n}\frac{T}{4}=\frac{c}{2n}\left(t_{D}-\frac{T}{2}\right)$$

is determined by the registration time $t_{D}$ and the pulse duration T, while the interval length $\Delta =\frac{c}{n}\frac{T}{2}$ is determined by the pulse duration T only. It is clear that the interval length Δ sets the spatial resolution for the described Rayleigh scattering sensing. 

In terms of the registration time $t_{D}$ (that then defines the interrogated fiber point $x_{D}$) Equation (5) is expressed as:

(7) $$I_{D}\left(t_{D}\right)=I_{1}\gamma^{2}\left(x_{D}\right)\int_{-\frac{T}{4}}^{\frac{T}{4}}\int_{-\frac{T}{4}}^{\frac{T}{4}}\exp\left\{-i2\omega_{0}\left(\tau -\tau^{\prime }\right)\right\}\rho \left(x_{D}+\frac{c}{n}\tau \right)\rho^{*}\left(x_{D}+\frac{c}{n}\tau^{\prime }\right)d\tau \;d\tau^{\prime }$$

Using new variables $\tau^{\prime }$ and $\Delta \tau =\tau -\tau^{\prime }$ Equation (5) could be transformed to:

(8) $$\begin{array}{cc} I_{D}\left(t_{D}\right)= & I_{1}\gamma^{2}\left(x_{D}\right)\int_{+\Lambda }^{\frac{T}{2}}\int_{-\frac{T}{4}+\frac{\Delta \tau }{2}}^{\frac{T}{4}-\frac{\Delta \tau }{2}}\exp\left\{i2\omega_{0}\Delta \tau \right\}\rho \left(x_{D}+\frac{c}{n}\left(\tau -\frac{\Delta \tau }{2}\right)\right)\rho^{*}\left(x_{D}+\frac{c}{n}\left(\tau +\frac{\Delta \tau }{2}\right)\right)d\tau^{\prime }d\Delta \tau +\\ & +I_{1}\gamma^{2}\left(x_{D}\right)\int_{+\Lambda }^{\frac{T}{2}}\int_{-\frac{T}{4}+\frac{\Delta \tau }{2}}^{\frac{T}{4}-\frac{\Delta \tau }{2}}\exp\left\{-i2\omega_{0}\Delta \tau \right\}\rho \left(x_{D}+\frac{c}{n}\left(\tau +\frac{\Delta \tau }{2}\right)\right)\rho^{*}\left(x_{D}+\frac{c}{n}\left(\tau -\frac{\Delta \tau }{2}\right)\right)d\tau^{\prime }d\Delta \tau +\\ & +I_{1}\gamma^{2}\left(x_{D}\right)\frac{n}{c}\Lambda \int_{-\frac{T}{4}}^{\frac{T}{4}}\rho \left(x_{D}+\frac{c}{n}\tau \right)\rho^{*}\left(x_{D}+\frac{c}{n}\tau \right)d\tau^{\prime } \end{array}$$

where a typical length $\Lambda <<\lambda_{0}$ of an individual reflection center $\rho \left(x_{i}\right)$ is introduced to analyze the last term independently from the first two. 

Finally, Equation (6) is reduced to the following set:

(9) IDtD=2I1γ2xD∫ΛT2Reexp−i2ω0ΔτFΔτ, xDdΔτ+I1ΛF0, xD,

where

(10) $$F\left(\Delta \tau ,\;x_{D}\right)=\int_{-\frac{T}{4}+\frac{\Delta \tau }{2}}^{\frac{T}{4}-\frac{\Delta \tau }{2}}\rho \left(x_{D}+\frac{c}{n}\left(\tau +\frac{\Delta \tau }{2}\right)\right)\rho^{*}\left(x_{D}+\frac{c}{n}\left(\tau -\frac{\Delta \tau }{2}\right)\right)\;d\tau$$

**Figure 2:**
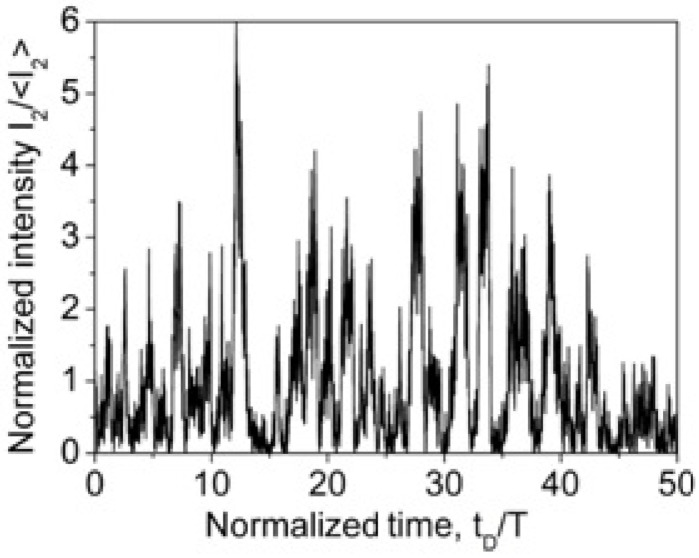
Typical time behavior of the simulated backscattered signal $I_{D}\left(t_{D}\right)$. The distribution of the reflection centers $\rho \left(x\right)$ is calculated as a delta-correlated Gaussian stochastic process with zero mean, for simplicity $\alpha \left(x\right)=0$ is taken.

Figure 2 shows the typical time behavior of the detected signal $I_{D}\left(t_{D}\right)$ as it is described by Equations (9) and (10). The distribution of the reflection centers $\rho \left(x\right)$ over the fiber length has been simulated as a delta-correlated in space Gaussian stochastic process with zero mean, i.e., $\langle \rho \left(x\right)\rho^{*}\left(x+x^{\prime }\right)\rangle \sim \delta \left(x^{\prime }\right)$, where $\langle \ldots \rangle$ means averaging over the fiber length and $\delta \left(x^{\prime }\right)$ is the Dirac delta-function. One can see that the intensity $I_{D}\left(t_{D}\right)$ demonstrates a pure stochastic behavior with a 100% modulation depth. Importantly, the simulated $I_{D}\left(t_{D}\right)$ traces are completely repeatable from pulse to pulse, as they are determined by the used distribution $\rho \left(x\right)$ and probe laser pulse parameters only. 

Although Equations (9) and (10) describe Rayleigh backscattering in a non-perturbated sensing fiber, they allow specific features important for an advanced understanding of the ϕ-OTDR sensing operation to be highlighted. 

1. A low fluctuated contribution to the detected signal $I_{D}\left(t_{D}\right)$ is expressed by the last term in Equation (9):

(11) $$I_{D0}\left(t_{D}\right)=I_{1}\gamma^{2}\left(x_{D}\right)\frac{n}{c}\Lambda \int_{-\frac{T}{4}}^{\frac{T}{4}}\rho \left(x_{D}+\frac{c}{n}\tau \right)\rho^{*}\left(x_{D}+\frac{c}{n}\tau \right)\;d\tau^{\prime }$$

It is an incoherent part of the backscattered signal that is commonly employed with a traditional optical time domain reflectometer (OTDR) based on a low coherent laser source. In such systems, the recorded signal reproduces the distribution of loss coefficient $\alpha \left(x_{D}\right)$ along the fiber length.

(12) $$I_{D}\left(t_{D}\right)=I_{D0}\left(t_{D}\right)\sim \gamma^{2}\left(x_{D}\right)$$

Equation (11) is valid as well if the distribution of reflection centers is strongly del-ta-correlated and uniformly distributed over the fiber length (i.e., $F\left(\Delta \tau ,\;x_{D}\right)\equiv 0$ at Δτ≠0). Importantly, the signal expressed by Equation (11) is not sensitive to a change of the probe laser frequency. It is also insensitive to a slight change of the reflection point positions, caused, particularly, by the effects of the external physical field.

2. Commonly, the distribution of the reflection centers is not strictly delta-correlated. The fluctuating part of the detected intensity is expressed by the first term of Equation (9):

(13) I˜DtD=2I1γ2xD∫ΛT2Reexp−i2ω0ΔτFΔτ, xDdΔτ

The function $F\left(\Delta \tau ,\;x_{D}\right)$ defined by Equation (10) is the autocorrelation function describing the distribution of Rayleigh backscattering centers. One can see that the detected signal is expressed as a Fourier component of the autocorrelation function $F\left(\Delta \tau ,\;x_{D}\right)$ accounted for the frequency of $2\omega_{0}$. Commonly, such a spectral component describes fiber Bragg grating with the reflectivity peak at $\omega_{0}$ possessing a periodic distribution of the refractive index along the fiber length with the spatial period of $\frac{\lambda_{0}n}{2}=\frac{n}{c}\frac{\pi }{\omega_{0}}$. Thus, the signal ${\tilde{I}}_{D}\left(t_{D}\right)$ could be thought of as an individual Bragg component extracted by the probe laser frequency from the energy spectrum (i.e., Fourier transform from the autocorrelation function) of reflection points distributed along the fiber. One can see that the signal ${\tilde{I}}_{D}\left(t_{D}\right)$ is very sensitive to fluctuations of the probe laser frequency $\omega_{0}$ and to changes of mutual positions of the reflection centers $∼\rho \left(x\right)$. This inherent sensitivity underlies the use of the ϕ-OTDR for distributed sensing. The effect of external physical fields (pressure, temperature, vibration) on the sensing fiber is similar to that on the fiber Bragg grating. The external perturbations affect the mutual positions of the reflection centers $∼\rho \left(x\right)$ that in its turn affect the signal ${\tilde{I}}_{D}\left(t_{D}\right)$.

3. Equations (9) and (10) have been derived for a non-perturbated sensing fiber interrogated by a single coherent pulse. However, they can be easily extended on a perturbated fiber periodically interrogated by coherent pulses one by one with the period W. When a sensing fiber is perturbated by an external field, the distribution of the reflection centers $∼\rho \left(x\right)$ slightly changes. To be more concrete, an external force stretches the fiber making the distance between the neighboring reflection centers slightly longer. Such an elongation of the fiber does not affect the incoherent part of the detected signal (see Equation (11)). However, it does affect the coherent part given by Equation (13). Mathematically it can be expressed as replacement $\Delta \tau \to \left(1+s\left(x_{D}\right)\right)\Delta \tau$ in Equation (9), where $s\left(x_{D}\right)<<1$ ($\sim 10^{-6}$) is a small parameter evaluating the effect of the external force on the fiber at point $x_{D}$. As the external force varies in time, it modulates $s\left(x_{D},t\right)$. Therefore, the signal $I_{D}\left(t_{D,m}\right)$ detected in the time moments $t_{D,m}=t_{D}+mW$ by the consequent probe pulses expresses modulation $\delta I_{D}\left(t_{D,m}\right)\sim s\left(x_{D,}t_{D,m}\right)$, where m=0, 1, 2… is the probe pulse number. Although the sensitivity of the signal modulation $\delta I_{D}\left(t_{D,m}\right)$ to the modulation of $s\left(x_{D,}t_{D,m}\right)$ drastically depends on the fiber point $x_{D,}$, one can conclude that a periodic modulation of the external force causes a similar periodic modulation of the parameter $s\left(x_{D,}t_{D,m}\right)$ that in its turn is converted to the periodic modulation of the signal $I_{D}\left(t_{D,m}\right)$ recorded with consequent probe pulses. It is clear that to this effect the external field modulation frequency should be much lower than the repetition rate $R=\frac{1}{W}$ of the pulse interrogation. In this case the processing of the signal $I_{D}\left(t_{D,m}\right)$ for different points $x_{D}$ along the fiber enables a distributed restoration of the acoustic spectra applied to the sensing fiber.

4. The response of the signal modulation $\delta I_{D}\left(t_{D,m}\right)$ to the modulation $s\left(x_{D,}t_{D,m}\right)$ drastically depends on the fiber point $x_{D,}$. In each fiber point $x_{D,}$ the signal modulation $\delta I_{D}\left(t_{D,m}\right)$ is in proportion to $s\left(x_{D,}t_{D,m}\right)$. The sensitivity factor between them could be obtained directly from Equation (9). One can see that the effect of the external field leading to replacement of $\Delta \tau \to \left(1+s\right)\Delta \tau$ in Equation (9) could be compensated by the simultaneous shift of the probe laser frequency $\omega_{0}\to {\left(1+s\right)}^{-1}\omega_{0}$. In other words, at the frequency ${\left(1+s\right)}^{-1}\omega_{0}$ the probe pulse in the perturbated fiber generates the same backscattered signal $I_{D}\left(t_{D}\right)$ as it generates at the frequency $\omega_{0}$ in the non-perturbated fiber. Therefore, the proportional factor $K\left(x_{D}\right)$ between the signal modulation $\delta I_{D}\left(t_{D,m}\right)$ and $s\left(x_{D,}t_{D,m}\right)$ could be expressed as:

(14) KxD=dIDtDds=−dIDtDdω0ω0=−4I1γ2xDω0∫ΛT2ΔτImexp−i2ω0ΔτFΔτ, xDdΔτ

**Figure 3:**
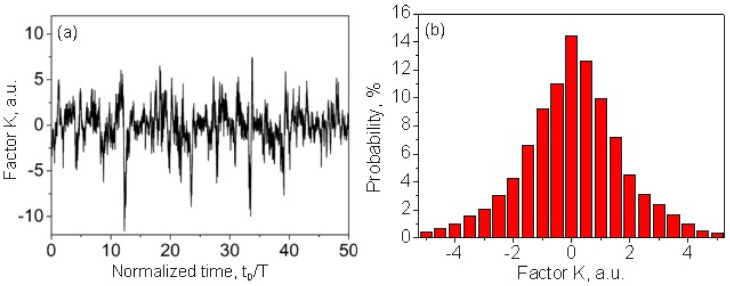
The sensitivity factor $K\left(t_{D}\right)$ (**a**) and its probability distribution (**b**) simulated for the case shown in Figure 1.

Figure 3a demonstrates the sensitivity factor $K\left(x_{D}\right)$ calculated for the same distribution of the reflection centers $\rho \left(x\right)$ as used in Figure 1. The sensitivity factor randomly changes along the fiber length. It can be positive or negative. One can see that there are fiber segments with very low sensitivity. Figure 3b shows a histogram describing the probability distribution of the sensitivity factor $K\left(x_{D}\right)$ that exhibits a typical Gaussian shape. The fiber segments with low sensitivity (<30% from an average $\langle \left|K\right|\rangle$) are found to occupy ~14% of the whole fiber length. It makes the absolute measurements of the external physical fields with the simplest ϕ-OTDR sensing analyzer rather questionable.

5. By the way, several advanced techniques for absolute measurements of the external physical fields with the ϕ-OTDR sensing have been demonstrated. In particular, for very slow changing or steady-state external fields (such as temperature heating) such measurements could be performed with the described simple setup equipped by a frequency tunable probe laser source. The procedure of the measurement is reduced to the laser frequency scanning with the aim to find for different fiber points $x_{D}$ a laser frequency ${\left(1+s\left(x_{D}\right)\right)}^{-1}\omega_{0}$ that compensates for the effect of an external field on the signal $I_{D}\left(t_{D}\right)$. It is clear that this method is time consuming and therefore could not be widely used in practical applications.

In conclusion, for this section, we have given a simplified insight into the physical mechanisms underlying the operation of a ϕ-OTDR setup for sensor applications. The key system parameters responsible for the distributed sensor performance are listed and their interconnects are highlighted. A number of effects associated with the ϕ-OTDR sensing are demonstrated and explained, in order to support a better understanding of the ϕ-OTDR techniques outlined in the rest of the review.

### 2.3. Quasi-Distributed DAS Using Fiber Bragg Gratings (FBGs)

DAS have experienced a steadily expanding transition into commercialization due to the enormous potential of DAS implementations in a wide range of application areas (cf. Section 3). Still, reaching the desired SNR level is highly challenging in long-range DAS systems relying on an inherently weak Rayleigh backscattering signal (RBS). Besides, the random nature of Rayleigh scatterers results in the occurrence of ‘fading points’ or dead zones (i.e., a dramatic decrease in sensitivity at random positions distributed all along with the sensing fiber). 

Therefore, sensitivity improvement for DAS is an active research area in which a paradigm change has recently been brought about [64]. The main idea of the new approach is to modify the sensing fiber itself rather than implementing hardware or signal processing enhancements on the interrogator side. The modifications on the sensing fiber can be implemented in either a distributed (e.g., UV grating inscription [65], femtosecond laser micro-machining [66], producing Rayleigh scattering defects by ultrafast laser irradiation [67]) or a *quasi-distributed* manner (e.g., dots by localized lased exposure [68], use of weak FBGs [69]). 

In this subsection, the specific group of quasi-distributed configurations that are based on weak fiber Bragg gratings (acting as scattering centers with known reflection coefficients and positions) is handled (the readers may refer to [64] for a detailed overview of the recent advances in FBG-assisted ϕ-OTDR technique).

**Figure 4:**
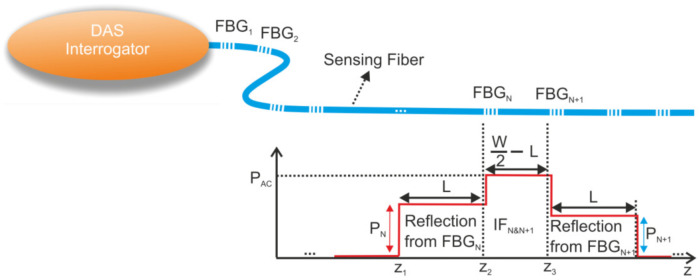
Scheme of a generic FBG-assisted DAS setup and theoretical power levels of reflected signals from a single FBG pair (FBG*_N_* and FBG*_N_*_+1_). L—distance between the FBGs. W—the interrogator pulse width.

FBG-assisted fiber-optic DAS techniques embrace various interrogation approaches [70,71]. Without loss of generality, we focus here on a generic setup as represented in Figure 4. It is composed of an optical time–domain reflectometer as the interrogator tool (e.g., ϕ-OTDR) and a sensing fiber where several weak (reflectivity values are typically around −40 dB), identical, equally spaced FBGs are inscribed. When a rectangular pulse of width *W* (probe signal) from a narrow-linewidth laser source is injected into the sensing fiber and under the condition that *W* is greater than twice the separation *L* between two successive FBGs, the signals reflected from both FBGs overlap on the ϕ-OTDR trace in a so-called *interference zone* (IF). 

Figure 4 schematically shows the ϕ-OTDR trace measured around a pair of FBGs (FBG*_N_* and FBG*_N_*_+1_). Three zones can be observed: two zones of length *L* corresponding to the parts of the signals reflected by FBG*_N_* and FBG*_N_*_+1_ that do not overlap and a zone of length *W*/2-*L* corresponding to interference zone IF*_N_*_&*N*+1_. This interference zone is created by the superposition of the electric fields ***E****_N_* and ***E****_N_*_+1_ (i.e., optical signals reflected from FBG*_N_* and FBG*_N_*_+1_). The complex reflection coefficient, **r***_N_*, and the complex transmission coefficient **t***_N_* for FBG*_N_* are determined as a function of the known parameters (i.e., grating length, grating pitch, and average refractive index modulation [72]). The power level corresponding to the IF*_N_*_&*N*+1_ region can be calculated as:

(15) $$P_{N\&(N+1)}=\left(E_{N}+E_{N+1}\right){\left(E_{N}+E_{N+1}\right)}^{*}={\left|E_{N}\right|}^{2}+{\left|E_{N+1}\right|}^{2}+P_{AC}\cos\left(\Delta \phi \left(t\right)+\theta \left(t\right)\right)$$

where *P_AC_* is the magnitude of the IF term (cf. Equation (16)), $\theta \left(t\right)=\arg\left(r_{N+1}\left(t\right)/r_{N}\left(t\right)\right)$ and $\Delta \phi \left(t\right)$ is twice the phase difference between FBG*_N_* and FBG*_N_*_+1_. This phase component ($\Delta \phi \left(t\right)$) contains useful information as it will be modulated when a perturbation is applied on the fiber section between FBG*_N_* and FBG*_N_*_+1_ [73]. Note that the distance between two successive FBGs determines the spatial resolution of the sensor system.

#### 2.3.1. Probe Pulse Configurations: Single (Long) Pulse versus Double (Short) Pulse Interrogation

In the analytical representation of Equation (15), the Rayleigh backscattering signal (RBS) coming from the fiber section between the FBGs (more particularly, the RBS contribution to the interference zone) is ignored. For the FBG-assisted DAS, the RBS may be considered as noise and can be alleviated by injecting a probe signal composed of two pulses of short duration instead of using a single relatively long pulse [69,74]. When the time duration between the front and the rear pulses is carefully designed (so that the optical signal of the front pulse reflected from FBG*_N_*_+1_ overlaps with the optical signal of the rear pulse reflected from FBG*_N_*), such a double pulse signal acts as a moving interferometer (similar to the signals through the sensing and reference arms of a conventional interferometer). By implementing the double-pulse configuration, the efficiency of measuring an external perturbation has been reported to be significantly improved [75].

#### 2.3.2. Multi-Reflection and Spectral Shadowing Crosstalk

**Figure 5:**
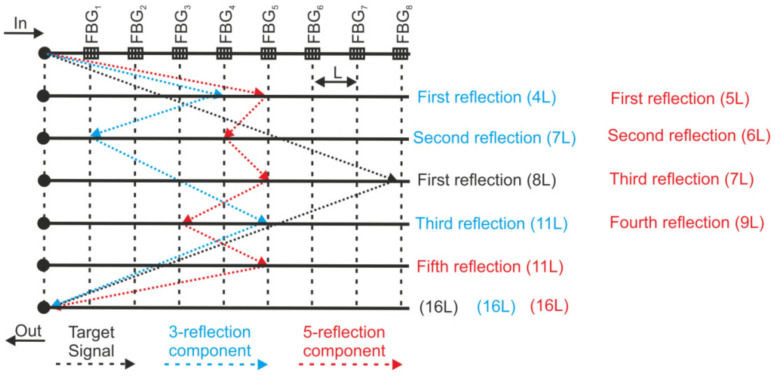
Schematic representation of multi-reflection crosstalk for an example case when a single-pulse reflectometer interrogates equally spaced FBGs. Possible 3-reflection and 5-reflection paths are superposed on the target signal from FBG_8_.

In designing FBG-assisted DAS systems, regardless of the probe pulse configuration, two crosstalk effects, namely multi-reflection crosstalk (MRC) and spectral shadowing crosstalk (SSC), should be carefully considered, as they potentially make the interpretation of the measurement results a challenging task. In addition to the optical signal reflected from a given FBG in the array, many other possible paths of interrogating the pulse exist which, after realizing multiple reflections (three reflections, five reflections, …), arrive at the interrogator at the same time as the useful signal (cf. Figure 5). 

Spectral shadowing crosstalk is another limiting factor when cascaded sensors sharing the same spectral characteristics are simultaneously addressed. Distortion occurs as the probe pulse passes all upstream FBGs (hence carries the spectral features) to reach a specific FBG. 

These two phenomena (MRC and SSC) have recently been interrelated and should be analyzed together in FBG-assisted DAS systems [76]. 

#### 2.3.3. Spectral Shadowing Crosstalk Mitigation

In Equation (15), *P_AC_* can be further developed to produce the concept of spectral shadowing. *P_AC_* can indeed be written as [71]:

(16) $$P_{AC}=2{\left|E_{in}\right|}^{2}{\left|T\left(t\right)\right|}^{4}{\left|t_{N}\left(t\right)\right|}^{2}\left|r_{N}\left(t\right)\right|\left|r_{N+1}\left(t\right)\right|$$

where ***E_in_*** is the electric field at the fiber input and ***T***(*t*) is the product of the transmission coefficients of all the preceding FBGs (FBG_1_ to FBG*_N_*_−1_). ***T***(*t*) depends on all the perturbations applied to the preceding FBGs. Therefore, if an applied vibration perturbs a previous FBG, the frequency content of this vibration will also be detected in the power level fluctuation of the interference zone IF*_N_*_&*N*+1_. This leads to a wrong diagnosis in the frequency content of the vibrations applied between FBG*_N_* and FBG*_N_*_+1_ (spectral shadowing crosstalk). In reference [71], a method to suppress the spectral shadowing in the case of the long pulse interrogation technique is proposed. It can be demonstrated as:

(17) $$\cos\left(\Delta \phi \left(t\right)+\theta \left(t\right)\right)=\frac{P_{AC}\left(t\right)-P_{N}\left(t\right)-P_{N+1}\left(t\right)}{2\sqrt{P_{N}\left(t\right)}\sqrt{P_{N+1}\left(t\right)}}$$

where *P_N_*(*t*) and *P_N+_*_1_(*t*) correspond to the optical powers reflected independently by FBG*_N_* and FNG*_N_*_+1_, respectively, as shown in Figure 5. By measuring the three power levels, it is possible to remove the contribution of ***T***(*t*) and, therefore, to suppress the spectral shadowing crosstalk. 

The spectral shadowing for the double pulse interrogation technique is less obvious since, in that case, only the interference zone is present on the ϕ-OTDR trace. *P_N_* and *P_N+_*_1_ do not appear if the distance between the two pulses is equal to 2*L* (which is the case in conventional double pulse interrogation techniques, since the signal reflected by FBG*_N_*_+1_ due to the second pulse fully overlaps with the signal reflected by FBG*_N_* due to the first pulse). Therefore, the compensation approach involving Equation (17) cannot be used. However, a simple method has been proposed in [77] to overcome this issue. The main idea is to interrogate the cascade of FBGs with a double pulse signal for which the delay between the two pulses has been reduced to 2*kL*, where *k* is the reduction coefficient (*k* ∈ [1-W/(2L),1]). This additional delay allows extra data on the ϕ-OTDR trace to be obtained: the signals reflected independently by each FBG (*P_N_* and *P_N+_*_1_) can also be measured (not only their interference), and Equation (17) can be used to suppress the spectral shadowing. 

The distributed time–domain acoustic sensors including the quasi-distributed FBG-based DAS-systems discussed above have passed from scientific setups in labs to become powerful measurement instruments. All recent advances improving the performance and accuracy of DAS systems (decrease in phase noise, polarization fading, and the above-mentioned spectral shadowing crosstalk mitigation, etc.) have resulted in the technical requirements for various engineering problems being met. Unsurprisingly, systems created by engineers have found widespread use in the technical sciences. The next section of our review is devoted to the application of DAS systems in the engineering sciences.

## 3. DAS in the Engineering Sciences (Andrey A. Zhirnov and Konstantin V. Stepanov)

Among the applications in engineering science, the following main areas can be distinguished: pipelines, railways, highways, and structures, including composite ones. These areas can be combined into two large groups: objects of great length (pipelines, railways, highways) and medium-range structures requiring permanent monitoring activity (composite materials in aircrafts). For the latter, DAS have replaced a large number of electrical point sensors that are difficult to implement due to the huge number of required electrical wires [78,79]. The following paragraphs will cover the main results obtained in the engineering applications over the past few years.

### 3.1. Pipelines

**Figure 6:**
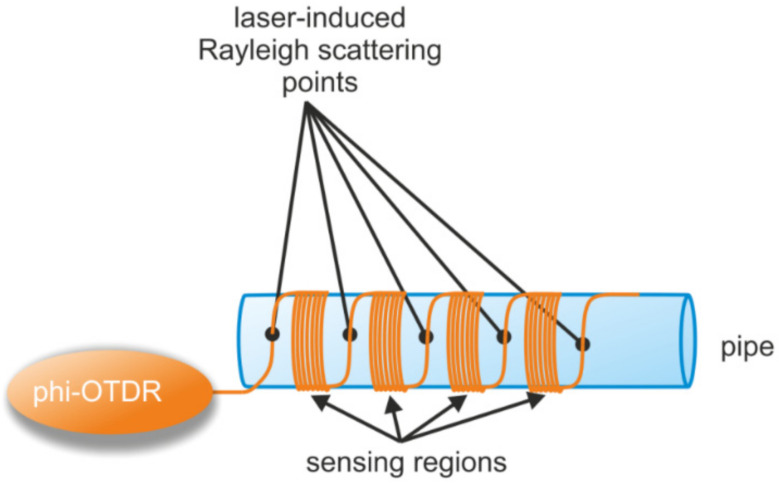
Schematic diagram of the DAS system for pipeline protection. Adapted from [83].

Pipeline monitoring has been one of the most common applications for distributed fiber optic systems (Figure 6). This is because they often pass through difficult to reach territories with no electricity supply. The rapid development of distributed fiber optic sensor systems and their installation in the field have made it possible to highlight the importance of not only improving the hardware characteristics of the sensor interrogator, but also the interpretation of the signals recorded by these devices. Recorded signals have been interpreted more and more by various machine learning methods, an overview of which can be found in the paper by J. Tejedor et al. [80]. 

The development of fiber drawing technologies has led to the development of using fibers with enhanced scattering points (dots) induced by a femtosecond laser or with reflective centers (weak FBGs) [81,82] (Figure 6). For example, DAS with enhanced scattering dots were used to record the potentially harmful effects on a pipeline of different materials (rubber, plastic, aluminum, and steel pipelines) with an average accuracy of 85% [83], proving their ability to identify various external impact events as authors used four hammers: soft rubber, hard-plastic, aluminum, and stainless-steel hammer heads. It was noted that the identifying error is larger at the branching of pipelines or where they are bent.

**Figure 7:**
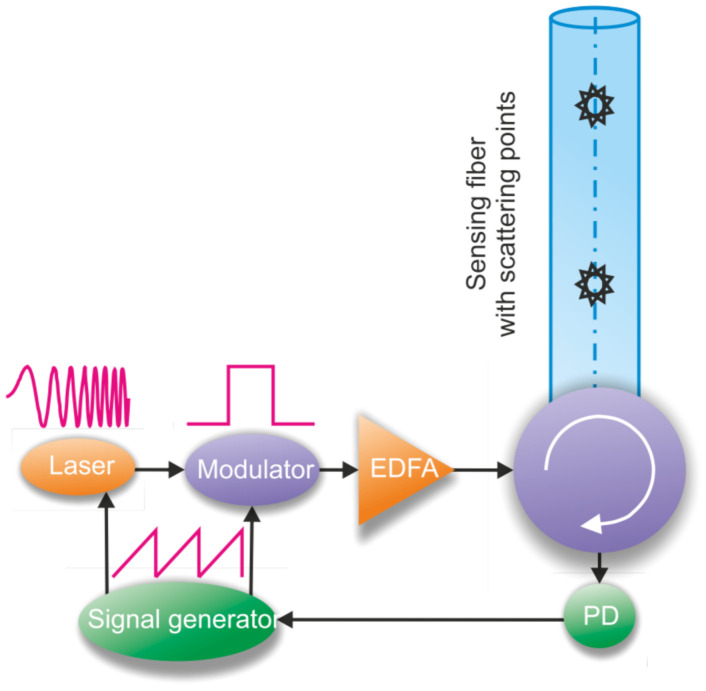
Optical scheme of the OFDR for absolute measurements. EDFA—Erbium-doped fiber amplifier, PD—photodiode. Adapted from [84].

In the work of a group from IRE RAS (Moscow, Russia) [84], an optical frequency domain reflectometry (OFDR)-based method (i.e., a linear chirp is introduced into optical probe pulses) for measuring absolute cable deformations (rather than a relative measurement) was presented, where special scattering points were placed in the sensor (Figure 7). In an experiment using 110 ns pulses, the deformation could be measured with a 0.16 μm at a 10 Hz repetition rate. The distance between the scattering points was set to 9.84 ± 0.04 m. This method could further be applied to real-time pipeline tracking systems. Current Brillouin optical time domain reflectometer (BOTDA)-based pipeline monitoring systems allow such measurements to be carried out only at intervals of about a few dozens of minutes.

**Figure 8:**
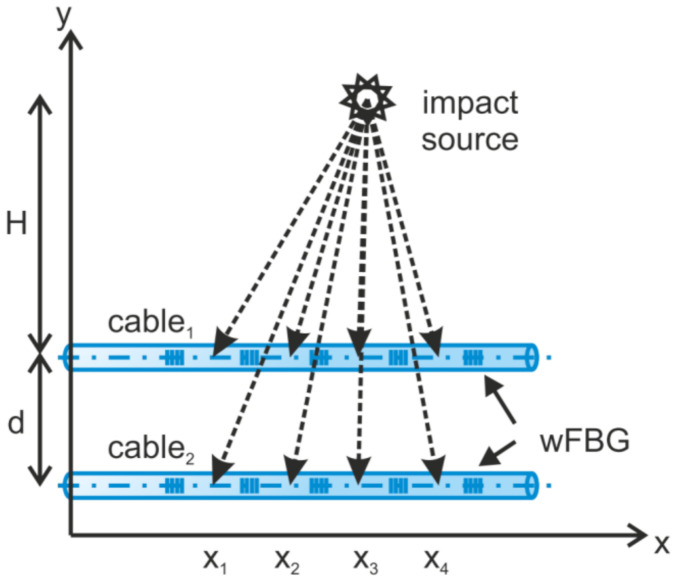
Sensing cable configuration for the measurement of the distance between the cable and the impact source. Adapted from [85].

The authors of [85] have presented the method for an impact source localization not only along the cable, but also away from it. To determine the location of the perturbation (impact source) and its distance from sensing fibers, arrays of weak-FBGs (wFBGs) inscribed in parallel sensor fibers were implemented (Figure 8). By using this method, a lateral distance (H in Figure 8) accuracy smaller than 20% was achieved in a maximum range of 25 m.

A pipeline integrity threat detection system was demonstrated in [86], where the use of several points on the cable for analysis was applied with fewer false alarms and an increased accuracy compared to the single-position approach for high energy events. In case of low energy impacts, this leads to a lower detection accuracy. It was also noted that for the correct operation, it is necessary for the analyzed signal to have an intensity higher than the background and the noise component. 

**Figure 9:**
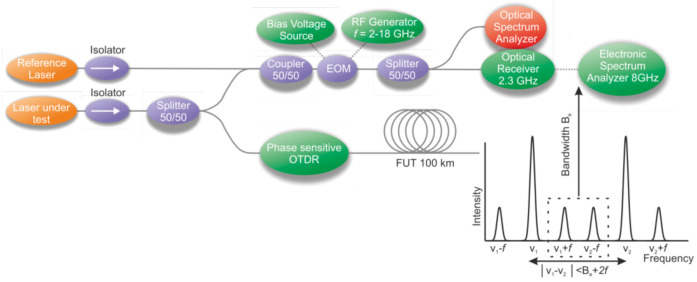
Schematic representation of the experimental setup for EOM-facilitated heterodyning measurements. The Allan variance and signal-to-noise ratio of Φ-OTDR signal can be determined for the same laser under test. Adapted from [88].

When the wFBG array is implemented in the fiber, the reflection level is increased to a level at least two orders of magnitude higher than that of Rayleigh scattering, resulting in an increased SNR, even in the absence of a preamplifier. In this case, it becomes important to study the phase noise of the laser source, which remains as the main noise contribution of the system [87]. The study of the noise of most popular laser sources was conducted by a group of researchers in [88], where a setup (represented in Figure 9) based on an electro-optical modulator (EOM) in a heterodyne scheme was assembled, which made it possible to compare the most popular sources in pairs using the Allan variance [89,90]. This characteristic describes the stability of the laser wavelength as a similarity of data part pairs taken with a different duration. The EOM added into the setup allowed the acceptable frequency difference between the laser pairs to be expanded. The sub-kHz OEW laser manufactured by “OE Waves, Inc.”, Pasadena, California, United States, was used as a reference for instantaneous linewidth measurements, and the frequency-locked CLA laser manufactured by “Wavelength References, Inc.”, Corvallis, Oregon, United States, was used as another reference to quantify the long-term frequency stability of a laser under test. The best couple of lasers showed an Allan deviation value at the level of the Lorentzian linewidth of 1 kHz in the time range from 10^−8^ to 10^−4^ s. As a continuation of this work, a fiber Mach–Zehnder interferometer with a path difference of 100 km with an acoustic optical modulator (AOM) located in one of the interferometer arms was used. This approach is easier to implement than the heterodyning configuration with a reference laser, where the optical frequency difference between the laser under test and the reference laser must be maintained within the acquisition bandwidth over the whole measurement time. The modified setup proposed in [91] allowed the stability measurement of sources in the range from 10^−8^ to 10^−1^ s. At the same time, the result was not affected by the low-frequency beatings of laser couples, which was manifested in [88].

The detection of leaks from holes of various shapes and the determination of the leakage volume using wFBGs has also been studied. The relationship between vibration frequency and pressure in the pipeline for holes of different shapes is presented in [92]. In this study, it was shown that the acoustic effect from a circular hole has a sharp start and then decreases, whereas the signal from a rectangular slit grows for 0.1 ms, reaches its maximum and then attenuates. Spectrum analysis of the signal resulting from a leakage showed a single high frequency signal peak at 186 kHz and a power spectral density in the range from 20 to 40 kHz in the case of the circular hole. The possibility of the operation under radiation conditions is also shown in [92].

In [93] a neural network was used to analyze the data signal, the layers of which were grouped into encoder, decoder, and classifier. The main advantage of this method is that it requires a smaller amount of labeled data compared to other types of neural networks analyzed therein. The main layers were 2D-convolutions (with pooling in the encoder and up sampling in the decoder) and specialties are the bi-directional long short-term memory (BiLSTM) layer and a self-attention mechanism at the end of the encoder block. The method made it possible to classify manual and machine excavations and the movement of vehicles with an average accuracy of 97.9% on the 88 km of the pipeline.

In [94] a combination of DAS and distributed temperature sensing (DTS) was considered to allow the random forest classifier to obtain an accuracy of 98.57 % in 6.79 ms based on the analysis of joint data from both systems. The system was used to detect leakages from pipeline.

### 3.2. Railways

Railways are particularly well suited for fiber sensing monitoring as a relatively high signal level is guaranteed by the enormous mass of the train. Fiber sensors can be applied to the entire infrastructure: tracks, bridges, adjacent territory (security), and trains. Commercially available fiber sensing solutions in this area already exist and provide a number of implementations on the railways, e.g., Frauscher Sensonic [95]. However, research for new and optimal algorithms for extracting information from the received signal are still needed.

The work in [96] is a good example of these research activities. It was taken into account that the DAS sensitivity (minimum level of impact amplitude) is interrelated with the registration accuracy (correct detections and false alarms rate). A neural network built on convolution and long short-term memory (LSTM) layers was trained based on the balance of these parameters. Signal sections with a specially selected time division were used as the input for the neural network. An accuracy of 8.0% false alarms and 85.6% correct detections was achieved. 

One of the leading teams working on fiber-optic sensors for railway monitoring are from the Technical University of Graz, where an introductory feasibility study [97], followed by a complete analysis including many infrastructure parameters [98], were realized.

**Figure 10:**
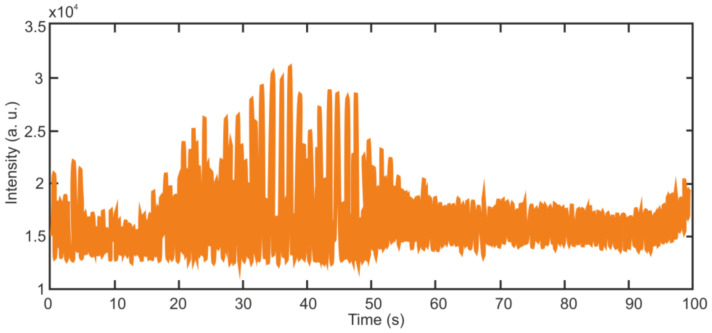
Rayleigh backscatter of a train ride. The peaks from 30 to 48 s represent wheel pairs passing a sensor point. Original obtained data.

Based on the abovementioned papers, the main difficulties of DAS-based railway monitoring systems can be defined as the limitations imposed by the railway construction requirements and the proper installation of optical cables. There might be situations when the cable is not laid in parallel to the tracks or too far from it. Such situations will lead to errors and blind spots. Nowadays DAS for railway applications remain a perspective technology that may replace many sensors such as axle counters by processing signals such as the ones demonstrated in Figure 10. However, the development of unambiguous algorithms is still needed to give more exact results, otherwise potential security gaps are generated.

### 3.3. Highways

Issues dealt with in relation to highways are quite similar to those of railways, but they have a higher complexity due to the random nature of the traffic. On highways, there is a variety of moving vehicles (in terms of weight, speed, and position on the road) and more noise, including the activity of pedestrians. Sensor systems can be extremely relevant (e.g., as a source of information on traffic congestion) when creating “smart cities”. 

The cable position with respect to the road and the dependencies of the system quality on the cable laying options are considered in [99]. The results of a two-year test of a system on a small section of urban highway, presented in [100], reported accuracies of 70% and 95%, for the classification of car types and their speed, respectively.

With the help of DAS, it was possible to observe global changes in traffic congestion associated with the COVID-19 pandemic (a decline in the congestion of all roads was registered, except for the roads going to the hospital), as presented in a cooperation work of scientific groups from Stanford University and Royal Observatory of Belgium [101]. Moreover, these data correlate with the data of cellular companies. The influence of high and low traffic congestion on various impact frequencies from 3 Hz up to 30 Hz is shown. 

In [102], based on a consideration of the structural similarity of the signal and the use of a convolutional neural network, a method that effectively increases the peak SNR when analyzing data with a 100 ms window was proposed. Such a small window duration allowed reactions to quickly moving events, which is important for highways. 

In [103], the application of the Hough transform for the search of car tracks on the waterfall (3D-plot of reflectogram sequence) data of a phase-sensitive OTDR is considered. The algorithm was tested on the data obtained from a telecommunication cable laid along the route. An accuracy of 73% was achieved in the registration of vehicle passages. The maximum number of errors was reported to occur in the registration of passenger cars. It can be deduced from the reported results that the recognition accuracy on highways is lower than on railways, leaving room for improvement in this area.

In addition to highway monitoring, the possibilities of more detailed tracking that includes the movement of people using fiber sensors are also being explored. As an example, a sensor with directed scattering centers recorded the movement of one and two people walking and running in different shoes in work by Z. Peng et al. [104]. Machine learning met both human identification and locomotion recognition requirements, with an accuracy of over 76.25% when using supervised and 77.65% when using unsupervised machine learning algorithms.

### 3.4. Structures

In addition to the abovementioned ultra-long-distance systems, in which distributed acoustic sensors are used all along the available sensor length before a critical signal decrease (amplification methods can increase the maximum length), DAS are also used in systems with a total fiber length of less than 10 km, especially when the compactness or explosion safety features of the sensor are important.

**Figure 11:**
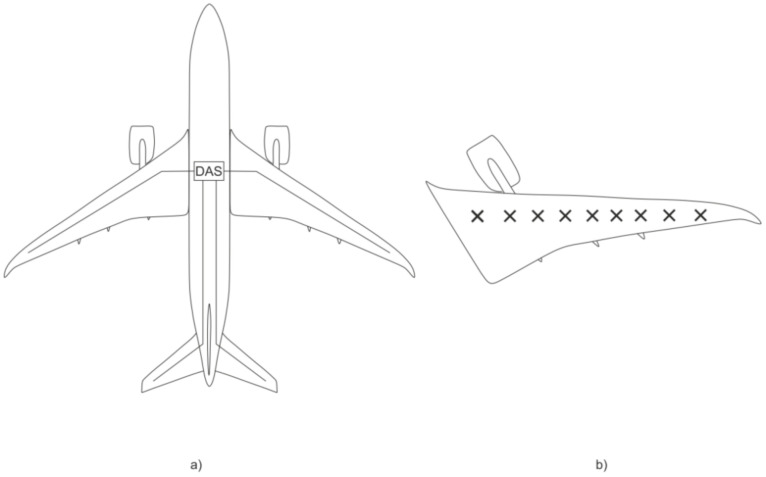
The used DAS-system (**a**) and the locations of accelerometers and strain gauges on the surface of the wing (**b**) for the verification of flutter frequencies. Adapted from [106].

Applications related to the composite materials are particularly important as the sensing fibers without additional coatings can be inserted between the layers during the piece’s construction without additional fixing. Such sensors allow material delamination to be registered at the early stages, before the entire structure is brought to a dangerous state. The critical issue of such implementations is the complexity of assembling a structure with built-in fibers [105]. There are control options for external sensor placement, as this is easier to implement. In [106], DAS measurements were carried out on a Boeing 747 in 1/26 scale in a wind tunnel. The natural vibrations of the aircraft structure (Figure 11) were measured with the frequencies of 5.1 Hz and 7.8 Hz, which coincided with the values measured by the traditional (accelerometers and strain gauges) sensors. Figure 11 shows the four sensing cables and the DAS system within the cabin (Figure 11a), as well as the array of accelerometers and strain gauges on the surface of the wing (Figure 11b) for the verification of the flutter frequencies. During the study, flutter frequencies of 5.1 Hz and 7.8 Hz previously detected by the DAS system were successfully confirmed.

Another application is the tracking of aircraft movements at airports. The test results were realized on an 8.1 km sensor, which was buried orthogonal to the runway of aircrafts at a distance of 4.2 km. The registration of a flying aircraft was reported by a scientific group from Beijing Jiaotong University in [107]. The 5 Hz region was shown as the most meaningful frequency range. 

**Figure 12:**
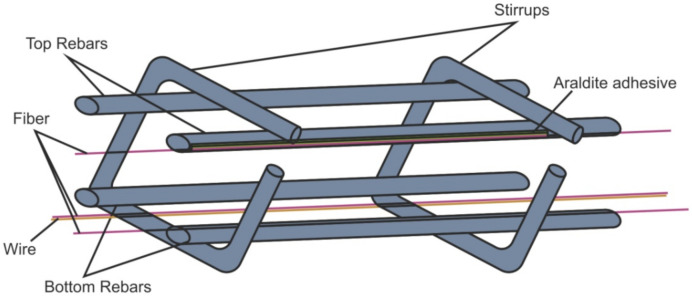
Fiber amid the stirrups. Adapted from [108].

Structural inspection is not limited to composite materials. For example, in [108], the placement of a sensor on a reinforced concrete structure is shown (Figure 12). With the help of DAS, it became possible to register critical bends and cracks at an early stage before they became visible to the naked eye and began to be a danger to the structure.

**Figure 13:**
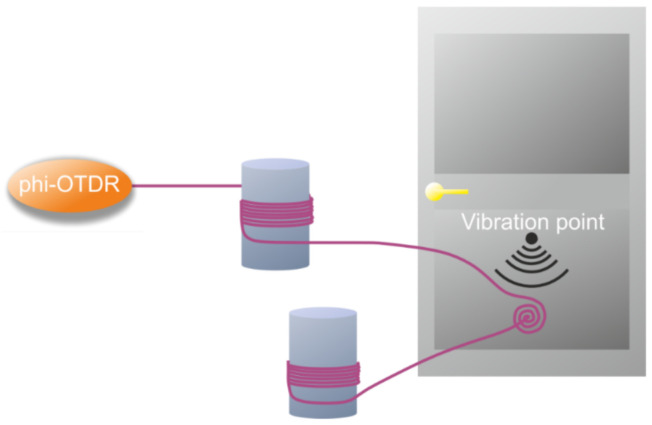
Scheme of a ϕ-OTDR sensor installation for window monitoring. Adapted from [110].

An overview of structural health monitoring using DAS was carried out in [109], which described applications for bridges, tunnels, pipes, geotechnical structures, and wind turbines. Some unusual applications of DAS can also be found (Figure 13), e.g., for window glass monitoring [110]. The experimental results showed that the average recognition rate of the model for eight kinds of vibration events within the glass window was 93.3%.

Another application is related to wind generators where a φ-OTDR makes it possible to measure the natural frequencies of the tower oscillations. Based on their amplitude, it is possible to draw conclusions about the reliability of the fastening (losses and breakages of bolts are possible) [111]. 

### 3.5. Other

**Figure 14:**
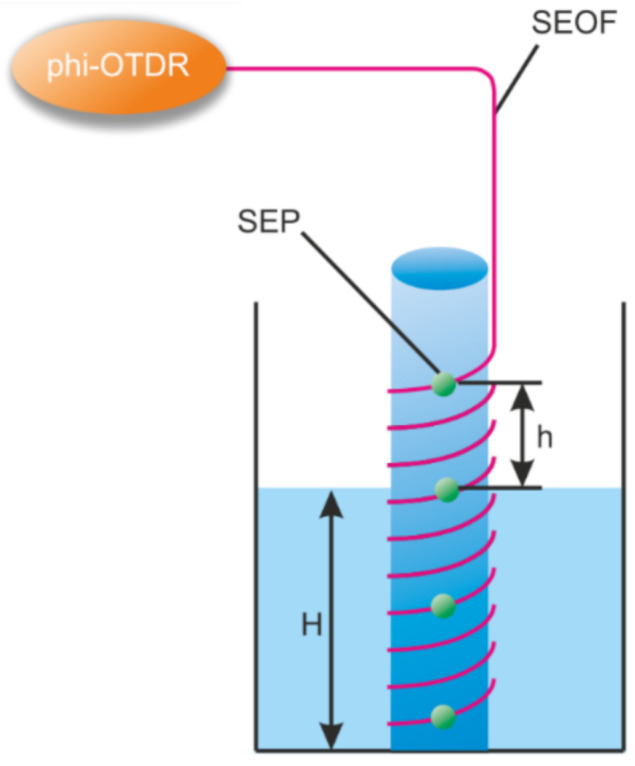
Schematic diagram of the distributed liquid level sensor based on the φ-OTDR system. SEP—scattering enhanced points, SEOF—scattering enhanced optical fiber. Adapted from [112].

In addition to the listed and more common applications of the distributed sensors, there are some original applications. For example, in the work of researchers from Huazhong University of Science and Technology [112], the level of liquid in a container was measured using a coiled fiber interrogated by DAS (Figure 14). The insufficient spatial resolution of the sensor was compensated by a small coiling step. The actual coordinate of the air–water interface was determined by the highly different level of signal instabilities from the areas located in liquid and air, with a difference in water and room temperatures of only 1 degree. The best reported spatial resolution was 142 μm in the range of 320 m without quality degradation.

**Figure 15:**
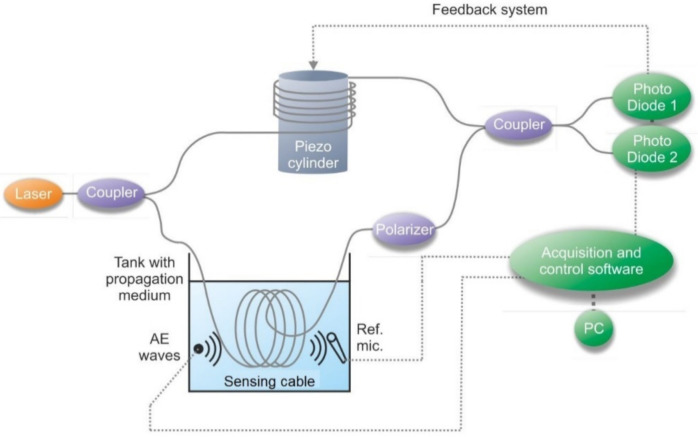
Schematic configuration of the all-fiber homodyne Mach–Zehnder interferometer and acoustic test bench. AE waves—acoustic emission waves, PC—personal computer. Adapted from [113].

An interesting part of the study of DAS is the analysis of the behavior of the sensor according to the used fiber optic cables. Increasing the acoustic sensitivity of the cable is a new direction which is particularly important for novel DAS development. Research about the SNR of DAS for cables in a tank with a propagation medium of various cross-sections is presented in [113]. Individual measurements were carried out on cable sections placed in a tank filled with water and sand (Figure 15). It was shown that the cable filled with hydrogel was more sensitive to acoustics than without it.

Another research group from Corning Inc. and OptaSense carried out field trials involving the cables buried at the test site [114]. In this work, the signals from a moving away acoustic impact source were recorded at an oscillation frequency of 86 Hz. 

Research data show that the cross-sectional configuration [113,114,115,116,117] and the cable placement method [118] in the ground affects the acoustic sensitivity of the cable and the overall system.

This section has presented some of the applications of DAS systems in the engineering sciences. However, the review would be incomplete without mentioning one of the most outstanding DAS engineering applications: geophysical measurements. These studies cover such a significant number of scientific works that this required the creation of an individual chapter. So, the next section is devoted to the application of distributed acoustic sensing systems in geophysics.

## 4. DAS in Geophysics (Boris G. Gorshkov)

### 4.1. Vertical Seismic Profiling

The unique properties of DAS attracted the attention of geophysicists shortly after the development of measurement systems with a good enough spatial resolution and sensitivity level while having the ability to reconstruct the phase and amplitude of acoustic signals. The first application was related to vertical seismic profiling (VSP). The sensing optical cable is placed in the exploration well in the same way as the seismic cable in traditional technology. This eliminates the presence of electronics in the well, which must operate at high temperatures and pressures, often in an aggressive environment. These conditions determine the high cost of traditional seismic instruments and entail significant operational expenses.

The advantage of the new technology is that the well is explored along its full depth in a single measurement. There is therefore no need for the sequential movement of the seismic sensors’ streamer along with the depth of the well, which necessitates the precise positioning of the streamer, ensuring reliable mechanical contact with the environment, accurate synchronization of all processes in time and, ultimately, a significant waste of time to carry out the research. When using DAS, it is enough to run the cable into the well, ensure its connection with the studied environment (for example, by cementing the well), implement the appropriate seismic action (for example, a near-surface explosion), and record seismic waves for a short time (usually a few seconds). The resulting recording of seismic signals allows P-S-, reflected, and converted waves to be identified and generally replaces traditional VSP surveys. At the same time, an optical cable is cheap and can be considered a consumable material; there is usually no need to remove it for reuse.

The first mention of the use of DAS for VSP in the literature appeared about ten years ago [119]. In this case, the relevant VSP data were demonstrated using the heterodyne DAS scheme developed by Schlumberger [120] and the direct detection scheme used by Silixa, Ltd (United Kingdom). [121].

The excitation of seismic waves can be carried out both by impulse sources and vibrators, with the help of which it is possible to generate mainly P- or S-waves, which provides additional information when interpreting the research results.

**Figure 16:**
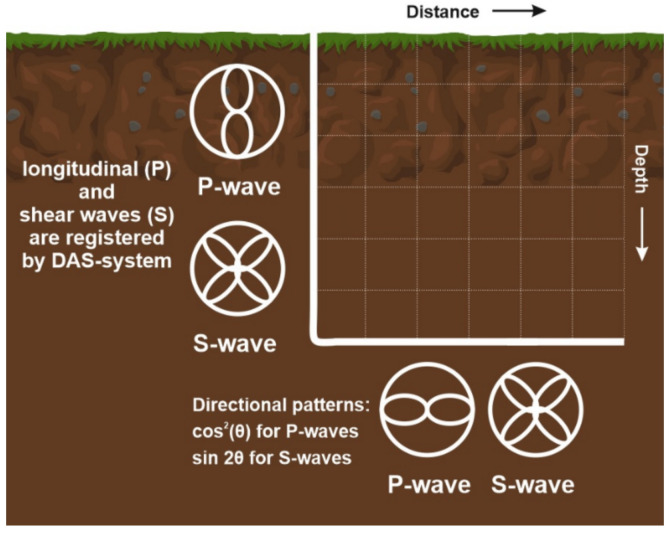
Longitudinal and shear waves in an optical fiber. Adapted from [122].

In contrast to three-component geophones, an optical cable of a conventional design has only one (longitudinal) axis sensitivity. Directional patterns are described as a cos^2^(θ) response of the fiber to the incident angle of the seismic wavefield for P-waves and sin 2θ for S-waves (Figure 16). In this case, it is possible to register both longitudinal (P) and shear waves (S). These dependencies have been confirmed experimentally [122].

This work also compared seismic data obtained using DAS with similar data recorded using traditional geophones. There was a good coincidence of both the kinematic parameters (the velocity pattern of propagation of various waves) and the dynamic (amplitude relationships). The reliability of the resulting DAS VSP data sets makes DAS technology an essential VSP acquisition tool.

However, in most cases, the signal-to-noise ratio for DAS at a comparable spatial resolution is lower than for traditional geophones. This is due to the small optical power of Rayleigh light scattering in low-loss fibers intended for optical communication. The use of special engineered optical fibers can significantly increase the magnitude of the backscattered signals, which leads to a noticeable improvement in DAS performance. For example, the authors of [123] demonstrated the waveforms recorded using DAS the quality of which was comparable to those obtained using geophones. 

In addition to increasing the signal-to-noise ratio, engineered fibers can improve the fidelity of seismic results, since in ordinary fibers, due to the randomness of Rayleigh scattering processes, distortions of both kinematic and dynamic parameters appear (the laws of this process are described in [124,125]). The use of specialty fibers with regularly arranged Bragg gratings allows such distortions and the fading phenomenon to be avoided.

An example of the use of specialty fibers with an increased Rayleigh scattering efficiency in DAS is given in [126], which shows an increase in the signal-to-noise ratio by 14 dB, compared to telecommunication fibers. It should be noted that such fibers are contained in OFS—A Furukawa Company (OFS Fitel, LLC, Norcross, GA, USA) product line.

Optical cables already installed in the wells for distributed thermometry can be used for monitoring. In most cases, they contain multimode fibers, while DASs typically work with single-mode fibers. It was however shown in [127] that the use of multimode fibers might even be preferable due to the possibility of avoiding signal fading, which is typical for the most straightforward of DAS setups.

### 4.2. Surface Seismics with DAS

Dozens of works have already been devoted to vertical seismic profiling, and it can be considered that this technology has already gained a foothold in industrial applications. Surface seismic is no less promising. At present, it is performed using discrete seismic sensors with relatively high characteristics. However, obtaining relevant data requires a very dense network of such sensors, which conflicts with economic considerations.

DAS technology provides an unprecedented opportunity for very dense coverage of the surveyed area with independent seismic recording channels at a meager price, determined by the lower price of the optical cable which can be considered as a consumable. Unlike borehole geophysics, the cable is located on the ground surface or at very shallow depths, replacing thousands of traditional sensors. Of course, the interrogators for DAS are quite expensive now, but they have a very long service life and a specific cost for any survey can be acceptable. As a result, seismic exploration can be performed quickly and economically [128]. 

In [129], the spectra of the phase velocity of surface waves were obtained, and it was concluded that reliable subsurface information could be obtained in just 100 s, when the seismic waves were excited by explosions in a quarry. The waves were recorded using the Stanford DAS Array [130]. An interfering effect of traffic noise near the optical cable was reported, however.

In [131], a study of the subsurface geological structure was conducted using DAS and dark optical fibers in an existing telecommunication cable near Sacramento, California. The sensitive area was 27 km long. Information on the velocity of surface waves was extracted using environmental noise (without particular sources of seismic waves). Maps of fine structural profiles and even the groundwater depth were obtained based on these data. In the same work, the possibilities of recording microseisms and teleseisms were demonstrated. In particular, eight earthquakes were registered in 2017–2018, both close and very distant (7757 km, Peru). The earthquakes were registered in the frequency range of 0.01–0.4 Hz.

While discussing surface seismic technology, it should be noted that the ordinary optical cables provide only one-dimensional sensitivity which is not always convenient. At the same time, a spiral cable option ensuring omnidirectional sensitivity was proposed [132]. Such a cable allows more detailed seismic information to be obtained [133,134], although it is expensive and less convenient to use due to its larger diameter (about 20 mm).

As already noted, the surface location of the optical cable makes it possible to register technogenic microseisms, and local and teleseismic earthquakes, including extremely low-frequency ones. In recent years, many researchers have been engaged in registering earthquakes using DAS [135,136,137,138,139]. 

The study in [140] is quite noticeable as it reports the characteristics of surface waves generated by lightning discharges. The arrival times of 18 events were registered to estimate the phase velocity of the near-surface, and the coordinates of lightning were obtained, which correlated well with the data of the National Lightning Detection Network of the USA.

The best surface seismic results are usually obtained with the underwater location of the sensitive cable using dark fibers in the seafloor communication lines [141,142,143,144,145] due to low level of environment noise.

In addition to studying the processes in the earth’s crust, including those associated with volcanic activity [146], DAS have begun to be used to control the dynamics of mountain glaciers and other natural hazards [147].

DAS technology can be used to study active faults on the seabed in addition to static measurements using Brillouin reflectometry [148].

Recently, works have appeared [149], where an underwater optical cable is used as a hydroacoustic array. Compared to traditional hydrophones, DAS-based technologies are inferior in sensitivity in the high-frequency region, and in the low-frequency region (below 10 Hz) they compete with piezoelectric sensors [149]. The advantage may be the simplicity of providing multiple measurement channels.

Thus, the use of distributed fiber-optic sensors can be considered as a new branch in geophysics [150]. 

In addition to research tasks, DAS is used to control hydraulic fracturing in hydrocarbon production [151]. It is possible to estimate the geometry and stimulated rock volume; this method is an excellent competitor to traditional methods of controlling hydraulic fracturing parameters. Finally, it has been shown that it is possible to monitor the fluid flow along the entire well [152], which is an essential task in oil production.

At the end of this section, it should be noted that the tasks of geophysics have become a powerful accelerator for the development of distributed acoustic sensors and for performance improvements. The high popularity of such studies has allowed researchers from related fields to draw attention to the unique features of DAS they can use in their own work. The next two sections of the review will describe such applications thoroughly. So, the following chapter will focus on the use of DAS in biology and other natural sciences.

## 5. DAS in Biology and Other Natural Sciences (Artem T. Turov)

The previous sections have demonstrated the various successful applications of DAS in geophysics, engineering, and material sciences. Many other fields of science and industry require efficient and highly specialized distributed acoustic sensors. Biology is one of them. Currently, the number of existing bio-acoustical sensors based on the DAS principle is very low compared to the number of potential applications.

**Figure 17:**
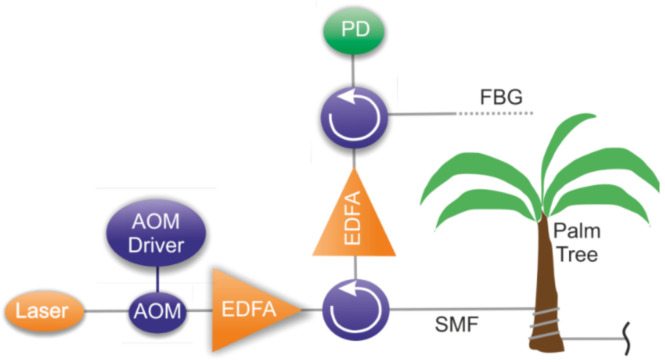
Schematic of the sensor system. PD stands for photodetector, FBG—fiber Bragg grating, EDFA—Erbium-doped fiber amplifier, SMF—single-mode fiber, AOM—acoustic optical modulator. Adapted from [153].

A research group from China and Saudi Arabia reported a successful red palm weevil detection with DAS [153]. The timely detection of weevils is vital as they can quickly eliminate the whole palm farm, while palm trees are valuable nutrition providers for many people. Already existing methods (e.g., visual detection, X-ray tomography, trained dogs) are either unable to perform early detection or cannot be adapted for several reasons: they lack scalability and have low data acquisition speeds. So, it turned out that DAS perfectly matched such monitoring requirements as they are quite easily scalable, non-invasive, and utilize non-ionizing radiation. The presented setup requires the sensing optical fiber to be placed around the trees (Figure 17). 

**Figure 18:**
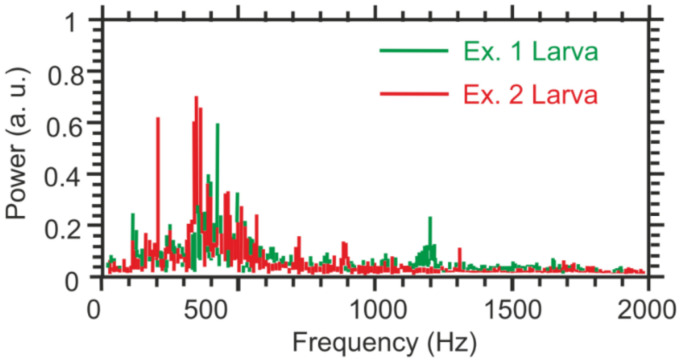
Typical spectra of sounds produced by 12-day old weevil larvae. Adapted from [153].

Firstly, the authors recorded a set of reference sounds produced by 12-day old weevil larvae using a high-quality pointwise recorder to train the neural network designed to detect the activity of these insects. The FFTs of two these recordings made in a row are presented in Figure 18. This figure also shows that the obtained frequencies could be easily detected using commercial state-of-art DAS systems, so the spectra obtained with developed DAS looked similar. During the training data recording time, the larvae were eating and moving naturally within the trunk without any restrictions. 

In such applications, distinguishing the signals from the perturbation source (e.g., palm weevil) from noises such as bird sounds and wind can be realized with the help of machine learning algorithms. The DAS system presented in [153] provides a spatial resolution of up to 5 m (for a sensing fiber) and can detect red palm weevil sounds with a dominant frequency of 400 Hz. The spatial resolution in the scale of tree height could be much more precise and also depends on the fiber turn number per meter. 

Studies [154,155] have described the requirements and existing ways of acoustic insect detection, mentioning DAS as a prospective solution for such monitoring needs.

**Figure 19:**
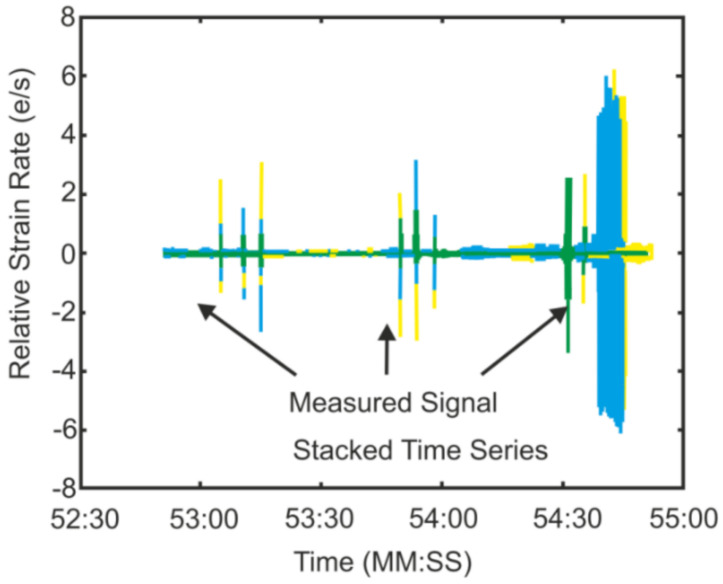
Detection of the 190 g weight dropping from the 41.5 cm height in a conditioning chamber at −70 degrees Celsius. Adapted from [157].

Biologists often need to detect, locate, and identify various animals in relatively large areas, as Browning et al. highlighted [156]. Due to their long measurement range, DAS can become powerful tools in those areas. As an example, Glaser et al. presented a successful demonstration of such a sensor for polar bear intrusion detection, which is extremely important for people living and working in the far north [157]. Figure 19 shows a stacked view of the channels in the DAS system that was used with relative strain rate on the y-axis and time on the x-axis. Experiments were conducted using a small environmental testing chamber for the measurements at temperatures above −70 °C. The study was performed to evaluate the functionality of the fiber at extreme temperatures. It was not intended to replicate field conditions other than the temperature, and as such the fiber remained spooled. A calibrated drop hammer was positioned on top of the test chamber as an acoustic and seismic source. A 190 g weight was dropped from a height of 0.42 m. Three sets of three temporally spaced impacts were performed at each test temperature. The authors obtained different strain rates for different temperatures within −30 °C and −70 °C and found them to be similar near 3 e/S (except −50 °C, where 9 e/S strain rate was detected). Figure 19 shows these results for −70 °C (the sequence of the peaks in the right is an external noise). Then, the authors tested their system in the northern region by using humans as an analog for polar bears and by calculating and comparing foot pressure in this initial study (the sensing fiber buried in snow at the depth of about 0.9 m was interrogated along a measurement range of 40 km at the temperatures around −70 °C).

The ability to detect footstep pressures of different values was studied, making it possible to classify polar bears of different kinds or distinguish them from other animals or humans. The sensor system was tested at the laboratory under *simulated* natural conditions (the polar bear intrusion was mimicked by people). Despite the fact that a human’s weight is less than that of a bear, human footstep pressure is practically equal to that of a small bear because the foot area of the latter is bigger. In addition, it was found that the external noise affecting the signal quality decreases with the decreasing temperature, so the SNR of the sensor working at temperatures below 0 degrees Celsius was better than the sensor functioning in normal SNR conditions. 

The authors of [158] have claimed that the early detection of animal invasions is crucial. The problem relates more to the invasion of biocenosis by some species that may be harmful to it, than to the direct danger for people. Still, this is extremely important because the threat of invasive species continues to increase, leading to the extinction of some other creatures. The authors suggested using a combination of acoustic and visual monitoring techniques for this kind of detection. The conventional acoustic sensing consists in general of an array of electronic microphones. It has already been mentioned in the sections above that though these devices are relatively cheap, their application is always connected with some disadvantages; therefore, they can most likely be substituted by DAS systems, at least in some instances, allowing the acoustic detection of some invasive species and classification of the intruder type. An extensive experience of the application of DAS in many spheres proves that their intrinsic advantages often offset their comparably high price.

As a severe consequence of global warming (melting glaciers), ice calving monitoring has recently drawn the attention of ecologists. Ice disruptions are usually detected accidentally by sailors or satellites, and their monitoring is a challenging task to perform due to the severe Arctic and Antarctic weather conditions. DAS are good candidates to implement in such an application. 

Anthropogenic factors and species invasion also affect various sea animals that also require specific monitoring systems. The existing systems still implement single or multiple electronic underwater microphones (hydrophones) [159,160]. 

Fiber optic underwater DAS were introduced about three decades ago and have gained popularity as a detector of intrusions, including ships, submarines, and divers. In 2021, Sandia researchers reported the first successful application of Silixa underwater DAS in the severe conditions of the Arctic Sea [161]. Although the principal objective of the research was the seismology of the seafloor, it was claimed that the sensor showed promising ice quake and whale songs’ registration results. The tested sensor allowed the actions as far out as 33 km in the ocean to be detected. This provides many opportunities to ecologists and biologists who need sensors of such kind.

Batelle states that DAS based on a standard optical cable are suitable for tracking animals (on large farms, safari parks, and wildlife sanctuaries, etc.) to better understand their behavior and needs. The ability of the proposed sensor to monitor weather conditions (wind, thunder quakes) was left for further investigations [162].

**Figure 20:**
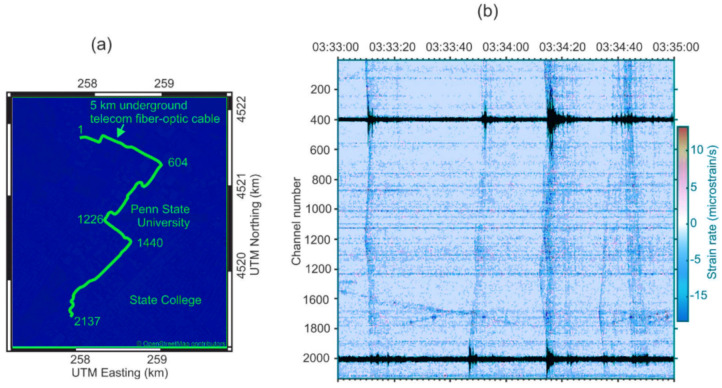
(**a**) Schematic representation of the optical fiber telecom cable used for this study; (**b**) Thunder quake detected. Adapted from [138]. The more precise colors are available in the reference paper.

In terms of measuring weather conditions and seismic activity detection, one of the most efficient utilizations of DAS was presented by scientists from Pennsylvania State University [138], where an already installed underground telecommunication cable and a commercial interrogator (Silixa iDAS2) were used (Figure 20a). The results of successful thunder quake detection were reported (Figure 20b), which may bring new perspectives to the investigation of the influence of the industrial vibrations on the mental and physical health of habitants. Organizing the sensing fiber line shown in Figure 20a, the authors of [138] planned to achieve a high resolution to detect small-scale subsurface features as well as sufficient coverage of the area and the increased likelihood of sensing near a feature of interest. They used existing telecommunication fiber paths, including at least two directions. The fiber line consisted of two fiber-optic sections spliced together, with a total fiber length about 5 km. These fibers are all underneath the Pennsylvania State University campus, and the study used a single set of unused fiber optics (dark fibers) in each fiber optic cable. These telecommunication cables were placed in buried concrete conduits at a depth of 1 m. The system allowed clear thunder events across the sensing line to be detected, one of which (occurred on 15 April 2019) is shown in Figure 20b. It presents 2 min raw recordings of six events between 03:33 and 03:35 UTC. The authors of [138] hypothesized that this recorded seismic energy was induced by thunder and/or lightning electromagnetic waves coupling to the ground to induce surface waves propagating in the shallow subsurface. The spatial variation could suggest the spatial attenuation of the shallow subsurface layer. The use of the system showed that the density of the obtained broadband DAS recordings provides extraordinary resolution that enables insight into their cause and allows one to distinguish between these various signals. It opens perspectives for its efficient utilization for weather and environment dynamics monitoring.

**Figure 21:**
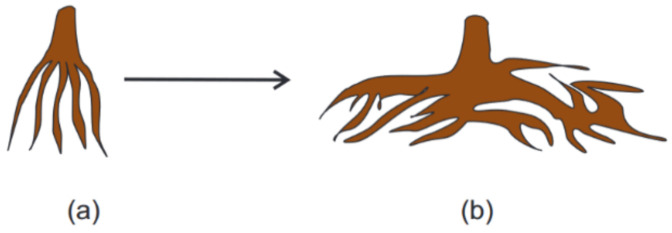
(**a**) Plants from the test group; (**b**) Plants from the control group. Adapted from [164]. See the photo in the reference paper.

It was believed for a long time that mechanical vibrations had no significant effect on flora. However, the studies carried out since the early 20th century and especially in the past few years have demonstrated that acoustic vibrations can have a negative [163] or positive [164] influence on the growth of plants. Researchers from Ivanovo State Power University [164], exposed the growing root of the plant to vibrations of a particular amplitude (0.5–1 mm) and frequency (10–15 Hz) once a day during a time interval of 6–8 h. These signals led to increased branch formation over a larger volume as represented in Figure 21a, compared to the plants from the control group (no vibration applied).

Plants from the test group bore fruit for 20–30 days longer than the plants from the control group, which led to a yield increase of 30–40%. Further development of such techniques in the future would ensure the formation of a well-developed, flat, horizontal root system in plants instead of a vertical one, which will allow them to absorb fertilizers, nutrients, and pesticides more efficiently, reducing the residue of harmful substances in fruits. The effect of the signal frequency ranging from 50 to 500 Hz on the plants, callus, and seeds was studied by Hassanien et al. [165]. The development of vibration ecology (techniques to obtain faster plant growth and increased yield) may be considered as one of the potential solutions to solve world food problems. The common problem of the abovementioned agricultural studies is that they provide only discrete knowledge about the influence of vibration on certain parameters on plants. So, to determine the effect of vibration with any possible amplitude and frequency, propagating in soil, the researchers need the distributed vibration-sensitive tool. DAS is one of the most promising solutions of this problem. However, for solving scientific problems and for design, distributed sensors are the optimal solution.

In summary, the application of DAS in biology and vibration ecology are expected to rapidly increase as more researchers will become aware of the fiber optic-based distributed sensing, its operation principles, and advantages. The perspectives of distributed acoustic sensors not only in biology and natural sciences, but also in arts, humanitarian sciences and culture are subjects of interest. In the next section, we will discuss how realistic and close these prospects are.

## 6. Acoustic Sensing and DAS in Humanitarian Sciences and Culture: Perspective (Yuri A. Konstantinov)

### 6.1. Fiber-Optic Acoustic Sensing in Humanitarian Sciences: Early Stages

In the 20th and 21st centuries, the cultural industry experienced an ever-growing demand for the development of audio information recording, storage, and distribution means. Despite the conservatism of the sound and music community, optical sensing technologies have also found their way into this industry. A good example is the successful commercial application of the simple optical sensors built on the principle of recording the intensity of the light beam emitted by the diode and reflected from the membranes placed at strings of guitars and bass guitars [166] by Christopher Willcox. It is surprising and reassuring that such a new technology found a place on the conservative market: it is rather challenging to find an innovative area in an industry where microphones and amplification equipment of the late sixties are considered the standard of the perfect sound. Nevertheless, such obvious advantages of optical pickup as a high signal-to-noise ratio and a sufficiently long sustainability, allowed musical instruments based on this principle to take an essential step towards performers and even find supporters among some locally famous artists.

**Figure 22:**
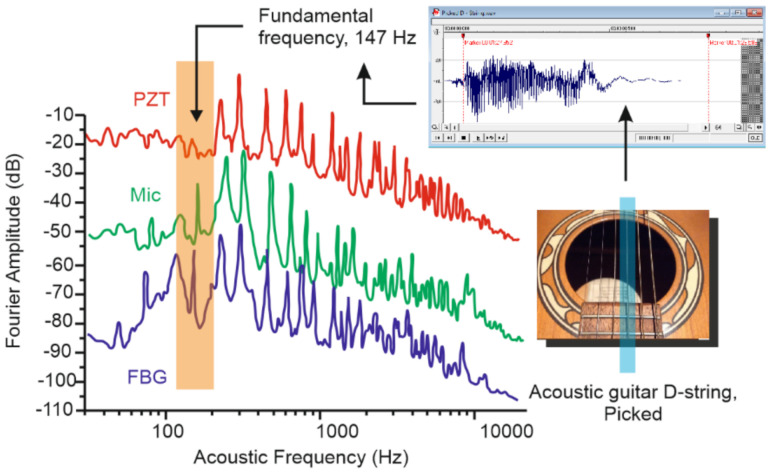
A spectrum of a picked acoustic guitar D3 string: recorded simultaneously with a FBG sensor, piezoelectric pickup, and condenser microphone. Adapted from [167].

Fiber optic technology has made its way into the audio industry. Back in 2009, fiber Bragg gratings were placed in solid and hollow-body guitar bridges and their signal was digitized and studied for processing suitability and musical aesthetics [167]. Loock et al. concluded in [167] that fiber Bragg gratings give a signal comparable in quality to traditional piezoelectric sensors (Figure 22). The figure shows the recordings with a plucked D3 string (146.83 Hz) which were undertaken using the narrowband FBG on one channel and either the PZT or the microphone on the other channel. The Fourier transforms display a high degree of correlation (all the FFTs in were offset for clarity), up to acoustic frequencies of about 12 kHz. Differences in the waveforms, especially with regard to the relative intensities, are readily attributed to the different positions at which the PZT and FBG were placed on the guitar. The used microphone was more sensitive to ambient noise and, in the described case, showed a noticeable signal below 100 Hz. The authors of [167] assumed that this frequency appeared due to computer cooling fans. It is clearly seen that frequencies that do not belong to the harmonic series of the fundamental string vibration also exist and sensed by the microphone, PZT, and FBG and this brings the coloration of the tone of the guitar. The diagrams cannot show how it really sounds, so the reader could refer to the sound files attached to [167]. 

The following instrument by Avino et al. [168] has Fabry–Perot cavities in the optical fibers as sensitive elements. In contrast to the previous work, the authors managed to obtain more exciting results in the sensitivity range of up to 25 dB and signal distortion when recording it in the dynamic range of up to 50 dB. In addition to the frequency range and other essential setup characteristics’ evaluation, the authors carried out another rather exciting experiment. They placed a musical instrument equipped with an interferometric sensor and a typical built-in piezo sensor, in front of the speaker, performed a linear sweep in a frequency range from 0 to 20 kHz.

**Figure 23:**
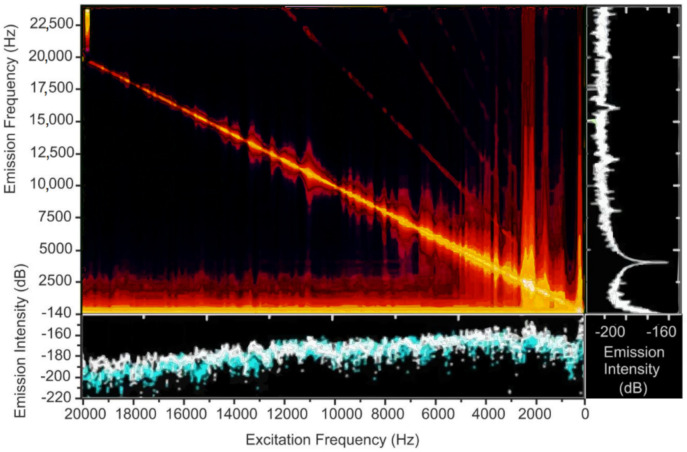
The excitation–emission matrix spectrum illustrates the feedback of the guitar’s soundboard to distortion from a speaker at different excitation frequencies. The color scale bar ranges from −220 to −180 dB. Adapted from [168]. The precise colors are available in the reference paper.

Figure 23 shows how extensively the array of data obtained from the embedded interferometric sensor are filled with specific overtones. In this experiment, a speaker was placed 20 cm from the guitar and emitted a frequency ramp at a rate of 1.9 Hz/s. The response of the PZT and FFP cavity transducers was acquired and processed by performing the Fourier transform of each 15 Hz interval. The obtained excitation–emission matrix (EEM) displayed emission response at the excitation frequency. Moreover, overtones of the excitation frequency are clearly seen, as well as a large number of resonances which depend on the guitar and the placement of the sensor on the soundboard. At these resonances the acoustic excitation causes the guitar body to vibrate at a broad range of frequencies near the excitation frequency, leading to a broadening of the emission spectrum. In the opinion of the authors of the review, this deserves a comprehensive study in the context of solving specific creative problems, for example, performing musical pieces that require guitar feedback from a combo amplifier. It is necessary to stimulate the development of Fabry–Perot sensors arrays interrogation with time and frequency domain reflectometry method in order to use this technique as part of a distributed acoustic recording system. Unfortunately, the authors of this review have not yet found such works. However, similar characteristics can be obtained by fiber Bragg gratings interrogation, which has become widespread in science and technology.

Among the pointwise sensitive elements, it is also appropriate to mention an amplitude fiber-optic microphone for concert and studio sound engineering [169], which allows permanent installation in large rooms, such as cathedrals, public or school halls, and sports halls. Made entirely of ultra-clear acrylic and suspended on its own clear fiber optic patch cable, it is practically invisible to the public. 

The unique design of the sensing element allows the frequencies required by the sound engineer to be transmitted, and the completely passive and non-metallic design opens up many other possible applications.

The examples of point-wise sensors given above were presented as a history reference. We believe that the positive experience of using single FBGs and other elements could give a reason for testing them as parts of a weak-FBGs-assisted DAS-system in the nearest future.

### 6.2. DAS in Sound-Engineering: First Steps and Perspective

The mentioned sources sufficiently demonstrate the ability of certain sensitive elements to perceive oscillations of acoustic frequencies emitted by musical instruments. Since the publication of references [167,168,169], many new quasi-distributed coherent OTDR sensor interrogation methods have appeared, reaching the characteristics close to the requirements of the specifications of modern sound recording devices. Such sensors can meet the needs for recording a large-scale musical and theatrical action and a unique, authentic sound in a particular room.

Traditional setups of coherent OTDR require significant modifications of both the interrogator, that is, the reflectometer itself, and the sensitive element (sensor) for use in detecting the sound of musical instruments and speech (singing). In a paper by Wu et al. [170], the authors demonstrated an increase in the system’s sensitivity after the location of some areas of the distributed sensor on a metal substrate after manufacturing a membrane. The authors of [171], Golacki et al., also mention the need to modify the sensor to apply a special polymer-metal coating. Both groups indicate the need to obtain the amplitude and the phase of the signal. The mentioned authors and the description of similar setups denote the problems of nonlinear response both in length and in frequency, which, according to [170], is in some way compensated by the use of fully polarization-maintaining apparatus by Qin et al. [172], and the use of a multi-frequency laser source, described by Zhou et al. [173]. The expansion of the signal along the fiber in traditional sound engineering can be compared to the mutual penetration of sound from different sources into different microphones, which can be both negative and positive when solving various creative problems and even mandatory for achieving the goal. An excellent example of such a case would be a recording of a classic drum kit, in which a kind of integrity of the material must be achieved [174]. In case of the negative influence of mutual penetration of sounds into recording channels, these issues are solved and can be solved at the stage of mixing in digital audio workstation (DAW) [175] by various traditional methods, including sound phase inversion. This is a very important aspect for sound production because the phases of sound waves have significant roles at sound mixing stage.

**Figure 24:**
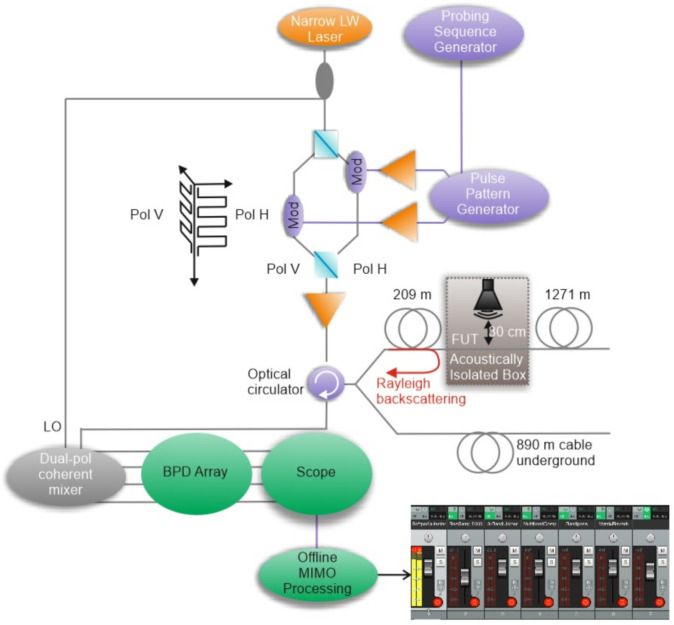
Schematic of the experimental setup. LW—linewidth, Mod—modulator, FUT—fiber under test, BPD—balanced photodetector, MIMO—multiple input multiple outputs. Adapted from [176].

The joint group from different institutions (Tomboza et al.) [176] recorded human speech by probing a coherent source with pulsed radiation in two polarization states, in both cases with different Golay sequences (Figure 24). 

**Figure 25:**
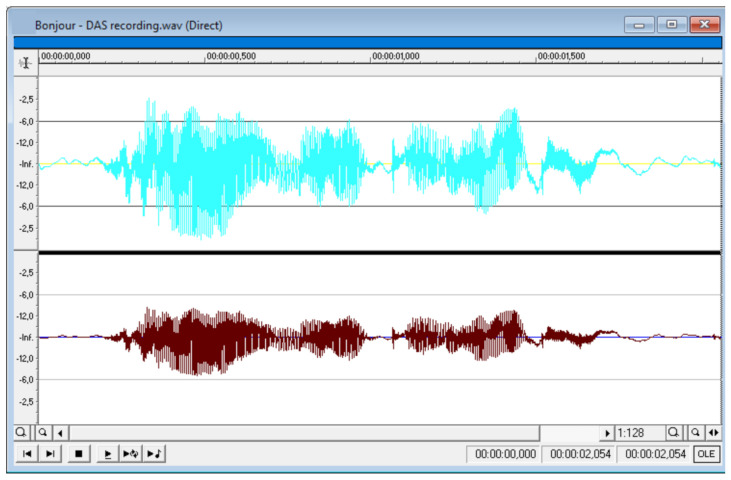
Blue line—the original signal of the human voice, saying “Bonjour,” brown color—the detected phase. The waveform is repeated by the review authors, please find the original waveform in [176].

Subsequent signal processing made it possible to read the word “Bonjour” from the local area of the distributed sensor with the upper recorded frequency of the order of 1.5 kHz (Figure 25). The result was also obtained using signal phase reconstruction using an optical hybrid. The upper frequency of 1.5 kHz allowed to distinguish only a single word, but for standard telephone signals the upper frequency of 3.4 kHz is required as a human voice has the overtones responsible for word recognition below this frequency. This study is a good first step for this field of research.

**Figure 26:**
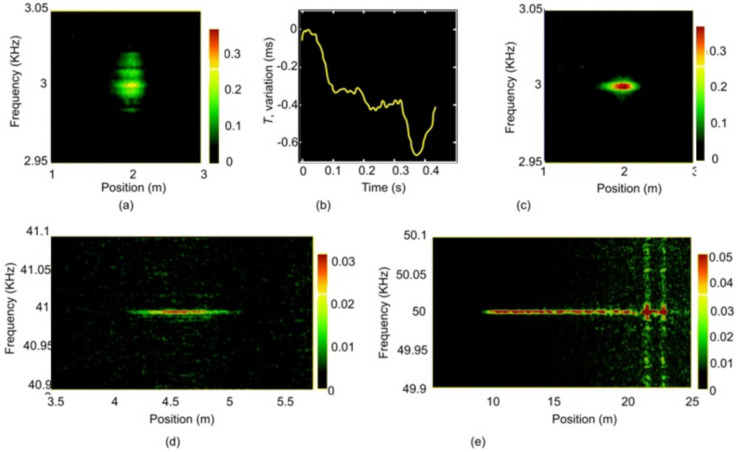
(**a**) Distortion spectrum before resampling; (**b**) Undesired sampling period variations; (**c**) Distortion spectrum after resampling; (**d**) Spectrum of a sinusoidal 41 kHz localized perturbation; (**e**) Spectrum of a 12.3 m long 50 kHz sinusoidal distortion. Adapted from [177]. The precise colors are available in the reference paper.

L. Marcon, A. Galtarossa, and L. Palmieri [177] found an unexpected and exciting solution based on a frequency domain approach. The development relies on an algorithm that provides a high acoustic bandwidth of dozens of kHz with a high spatial resolution of the order of several centimeters. The authors analyzed the setup imperfections and the phase noise impact on the measurements and experimentally demonstrated a method for their compensation. First, reflectograms were obtained in the frequency domain with and without exposure. In this case, a unique compensating interferometer was used, which monitors the nonlinearity of the radiation source tuning, as it happens in most commercial models of such devices. However, this approach meant that the coordinate axis of such reflectograms became nonlinear. To solve this problem, the authors proposed integrating a 3 kHz sound vibrations source into the hardware of a distributed acoustic sensor at specified locations, supposing that this will help collect data to eliminate distortions and build a more accurate picture using the resampling method. This made it possible to achieve impressive results in the distributed detection of frequencies, even beyond the audibility of the human ear—up to 50 kHz (Figure 26). To study the effects of the frequency sweep nonlinearity, a sinusoidal perturbation with a frequency arbitrarily set to *f* = 3 kHz was applied through a PZT at position *z* = 2 m of a 3 m long fiber. Figure 26a presents the initial spectrum of the retrieved perturbation without any processing. Instead of a clean peak, an evident spectral broadening with a 20dB-bandwidth of about 80 Hz can clearly be seen from the figure. An example of the undesired time sampling variations as function of the measurement time is shown in Figure 26b, and the perturbation spectrum after the resampling procedure is shown in Figure 26c, where a clean spectral peak at desired frequency of 3 kHz is clearly visible. Figure 26d demonstrates spatial accuracy of the system: the applied 41 kHz perturbation is spread over a length of 50 cm (most intense, ‘red’ zone) which corresponds to the effective spatial resolution achievable with the selected algorithm parameters. Figure 26e shows the possibility of monitoring a spread (12 m length) and high-frequency perturbation (50 kHz). The extracted signal is centered at the desired frequency and has a length of 12 m.

In modern digital sound engineering, recording such a signal and its digitization at such frequencies is quite essential—this excludes or makes controllable the appearance of various artifacts during further processing of the signal. The operating range of such systems is usually tens of meters, which would provide a high-quality recording of chamber ensembles or complex musical instruments, such as an organ relatively quickly. 

The group of Dr. Franciscangelis from the University of Campina, Brazil [178] reported on the creation of a real-time distributed optical fiber microphone, capable of handling a frequency-swept sinusoidal acoustic signal with the frequency up to 2000 Hz, music, and human voice. Though the quality of human voice recording is undoubtedly worse than the quality of original sound (the sound file attached to [179], obtained using the ϕ-OTDR, differs from the original one by the high frequencies and overtones deficit, while the meaning of the phrase might be understood), this sensor is still an innovation because no ϕ-OTDR-based microphone could perceive and transmit the disturbance in real-time before. As it is said, most of the previous inventions involving the ϕ-OTDR technique needed signal post-processing, while the proposed fiber-optic microphone can reproduce acoustic disturbance in real-time with the spatial resolution of 60 cm along the 300 m fiber section. This is possible thanks to the sample-and-hold electronic circuit, which is relatively cheap and straightforward.

Among other things, direct detection helps to simplify the signal-receiving part of the setup and decreases polarization fading. The records of music and human voice made with the proposed sensor are attached to the electronic version of the article. With proper development, this system can be used for concert sound engineering, as the outputs of such circuits can be switched with the inputs of mixing consoles.

DAS based on ϕ-OTDR setups helps overcome the disadvantages of more simple sensors, measuring the intensity of Rayleigh backscattering (i.e., based on standard OTDR technique). The microphone, presented in [179], cannot function in real-time, possessing the necessity to collect and proceed the data with a particular Matlab script, but it shows the possibility of utilizing a simple OTDR technique for sound recording. Although the mentioned software implements signal denoising, a 10 m section of optical fiber exposed to the acoustic waves has been spirally rolled to increase the sensitivity and improve the SNR. The obtained results show that the microphone performance is suitable enough for human voice detection (Figure 25). All in all, it can perceive the impact with the frequency from 300 to 3400 Hz on the 2.5 km optical fiber length with the spatial resolution of 10 m. This paper presents the reconstruction of the human voice, recorded with this intensity-measuring OTDR.

As seen in this section, the performance of distributed fiber optic sensors designed for recording vibrations of acoustic frequencies in the air and human speech, or musical instruments is getting better every year. In many ways, this rapid development is driven by the requirements in other areas, mainly oil, gas, geophysics, etc. It is safe to assume that in 7–10 years, an efficient distributed fiber optic sensor system for multi-track high-quality audio recording will be available on the market. The high price of this system will certainly be offset by its flexible and seemingly fantastic capabilities. To accelerate this moment, the sensory giants must cast their eyes on the hi-end audio market; and researchers working in the field of optoelectronics, even poorly experienced in music and sound production, must look at their daily scientific activities from a new creative angle. It is worth remembering that the existing distributed and quasi-distributed systems can already solve individual creative problems, and the issues and obstacles described above, in some cases, are not even challenging for them. For example, the highest notes of some instruments, even taking into account overtones, can be significantly lower than the required 20 kHz, which will effectively record a whole group of such instruments with a distributed acoustic pickup; in addition, not all musical instruments need to be recorded from a distance: for example, guitar resonators, cabinets, combo amplifiers, various string instruments, etc., which means that the distributed sensor has close contact with the vibrating surface, and there is no need to modify the coating or develop the membrane significantly. Finally, the dream of every sound engineer is to obtain an unlimited number of microphones covering as many spectral ranges as possible, becomes more realistic with the constant improvement of DAS technology. It could provide an amazing source data for DAWs, containing waves with different phases and spectra. The modern DAS systems have a significant number of the parameters desired by most sound engineers, but they still need to improve their spectral response linearity and signal processing time. The acquiring, processing, and the transmission of a single trace to the DAW within 20 ms will make possible DAS applications for live shows outside the studio.

In conclusion, there is another one thing certainly worth noting. Even though DAS systems, as just noted, are not yet perfect for live sound engineering, the recording of a rather big open-air music event using a distributed acoustic sensor has already taken place. This is evidenced by the work [138], already considered in the previous section of this review, where the existing telecommunication line was used for the acoustic monitoring of natural phenomena. The concert took place within the perimeter of the system. It turned out that the desired signal was modulated by the sounds of the concert, in which the well-known songs were easily guessed during playback. Their sequence matched the official track-list of the concert. We consider this success, albeit an accidental one, to be a good first step of DAS technology into the art.

## 7. Conclusions (Yuri A. Konstantinov and Ivan A. Lobach)

This literature review showed that distributed acoustic sensors, which are known to be efficient at intrusion sensing, structural health monitoring and railway supervision, are also being actively used for scientific research in geophysics, materials science, and other engineering disciplines. In addition, quite exotic applications of distributed acoustic sensors were investigated in relation to biology, urban studies, culture, and the media industry. 

Let us determine the main factors describing all mentioned scientific fields where DAS systems could be applied: 

1. The number of scientific papers related to the application of distributed acoustic sensors in this area;

2. The availability of technology for the given scientific industry;

3. The readiness of the technology for the given scientific field. It is determined by technical requirements in area of interest (see Table 1);

4. Assessment of development prospects (Integration to the area, see Table 1).

In geophysics, there are quite a few works devoted to the use of distributed acoustic sensors. According to the analysis of publications from journals of the Web of Science database, the geophysical application of DAS is one of the most widespread applications. The ResearchGate web platform also confirms this observation. This suggests that the publication activity in this industry is relatively high. Since these studies often find the support of governments, world corporations and large raw materials producers, including global businesses, DAS technologies are available for the industry, despite the high cost of the systems. The readiness of the technology for this scientific industry is also relatively high: this is confirmed by the presence of some high-quality commercial phase-sensitive devices. Considering that the geophysics industry poses more and more scientific challenges for distributed acoustic sensors, and scientific groups worldwide are engaged in their immediate solution, the rapid development of this technology in the industry in the coming years is ensured. However, the revolutionary overcoming of all the limits of the method described in the scientific literature at the end of the last century, most likely, will not take place in the coming years. Table 1 shows that the requirements for distributed fiber-optic acoustic sensors in the geophysics industry are quite well implemented. Special versions of DAS, created for recording ultra-low frequencies, confidently record the data scientists need. Basically, to obtain them, there is no need to use high bitrate analog-to-digital converters. Although there is no need for sensitivity to high frequencies to register geophysical data, such an option would be interesting for registering accompanying factors that affect a geophysical object: humans and/or animal activity and other natural phenomena. The data coming to the researcher from such a system can be displayed practically without delay due to the relatively low number of traces per second.

Things are a little different in the engineering sciences. Excluding such purely technical applications as perimeter security, monitoring of buildings, engineering structures, and other objects from consideration and considering only scientific work (research of new materials, including composite materials; study of the properties of new structures, etc.), it can be found that there is much less work using DAS technology than in geophysics. Despite the sufficiently high readiness of the technology for solving a whole class of research problems in engineering sciences, its availability for researchers is relatively not high. This can be explained, among other things, by the difficulties in acquiring a rather expensive system using grants from scientific funding. Nevertheless, since the technology is in demand, there is a task to reduce the system’s total cost by using less expensive components, so the prospects for DAS in this scientific direction are quite promising. Referring to Table 1 again, we can see that the application of systems in engineering disciplines does not have a well-defined range of desired frequencies, so it is set as broadly as possible. This is due to the fact that, for example, in such a discipline as structural health monitoring, it is important to register both long-period changes, close to static ones, and high-frequency vibrations, with frequencies exceeding tens or even hundreds of kilohertz. An 8-bit rate is sufficient for capturing the amplitude or phase of the measured signal for most applications in the industry. In technical applications, the speed of data processing and visualization can be important in the case of integrating a DAS system into a research or production cycle with control feedback, but we are not aware of such works yet. Therefore, the existing data processing delay for applications in this area can be called acceptable.

In biology and other natural sciences, the application of distributed optical sensors is just emerging. The first works of this kind are mentioned in our review; there are relatively few of them. The question of the readiness of DAS technology for this scientific industry is controversial since many different tasks can be set. For example, in the case of monitoring forest or marine flora and fauna, the method becomes difficult to implement from the point of view of data interpretation, which can be partially compensated by using quasi-distributed systems with weak reflectors in the line. However, it should be admitted that, according to the authors, there is not a single commercially available system, let alone inexpensive, for use in this scientific field. The life sciences industry does not yet have a clearly defined frequency range that DAS manufacturers should focus on. The researchers are currently using typical commercial systems available on the world market. Due to the variety of tasks and not yet high interest of biologists, it is rather difficult to determine the other parameters as well.

Nevertheless, the described sensors created for biological purposes turn out to be the cheapest opportunity for the monitoring demands they satisfy. Readiness and availability of technology are also not so high. With a decrease in the cost of systems, the prospects are also quite good.

The use of DAS in music, particularly in sound engineering and recording, is quite interesting. While there is almost no scientific information on the use of distributed acoustic sensors for this purpose, the review presents some works describing the studies of individual structural elements of quasi-distributed sensors—fiber Bragg gratings. Since the distributed acoustic sensors with a satisfactory amplitude-frequency response can record the desired frequencies from 20 Hz to 20 kHz and given the successful start of the research on single FBGs, the readiness of the technology for use in this industry is relatively high, which cannot be said about the accessibility. We will also evaluate the prospects provided that the cost of the system decreases as quite attractive. If we look at Table 1 again, it is easy to see that the desired frequency range is already covered by systems based on coherent reflectometry and frequency domain reflectometry. Despite the fact that the recordings that have passed the final mastering are digitized at a 16-bit bit depth (the so-called CD-quality), the raw track data is usually subjected to 24-bit or 32-bit quantization, thus reducing the likelihood of artifacts appearing at the signal processing stage. The problem of using high bit depths and, at the same time, high sampling rates slows down the integration of DAS technology into art. If it is possible to achieve such digitization parameters with a signal processing delay of the order of 20 ms, it will make distributed acoustic sensors more attractive to the industry.

To be applied in different areas, many of DAS-system parameters require significant improvement in performance, which can be achieved by decreasing phase noise [184], polarization fading [185], and improving the parameters of coherent sources [186,187] and their testing methods [84,85].

Summing up, it should be noted that such a powerful and flexible scientific instrument as a distributed acoustic sensor can and should be used more often in various branches of science, not only technical ones. The main obstacle in integration of this technology into various scientific fields is the high cost. In some cases, the DAS systems need the improvement of some parameters. If the world’s leading manufacturers make efforts to reduce the cost of the element base of their products and to improve custom parameters, this will help making an essential step towards expanding the range of DAS applications and obtaining scientifically exceptional knowledge about the world around us.

## Figures and Tables

**Table 1:** Area of interest requirements and the best achieved parameters of DAS.

Area of Interest, Requirements	Freq. Ranges	Digitizing Bitrate	Processing Delay	Integration to the Area
Geophysics	from 0.05 to tens of Hz	Usually 8 bits	from 0.5 s to few seconds	High
Engineering	From Hz to hundreds of kHz	Usually 8 bits	from 0.5 s to few seconds	High
Biology	Usually within 20 Hz–20 kHz	N/A	N/A	Low
Arts	20 Hz–20 kHz	24–32 bits	<20 ns for live recording, N/A for studio	Medium
Best achieved *	0.001 Hz [180]–80 kHz [181]	16 bits [182]	1 ms [183]	N/A

* It should be noted that the best achieved parameters are taken from various works and characterize different systems, therefore it is necessary to take into account that if any one of the system characteristics is optimal, it could lead to the decrease in other parameters.
